# Interferon-gamma ameliorates experimental autoimmune encephalomyelitis by inducing homeostatic adaptation of microglia

**DOI:** 10.3389/fimmu.2023.1191838

**Published:** 2023-06-02

**Authors:** Juan E. Tichauer, Gabriel Arellano, Eric Acuña, Luis F. González, Nirmal R. Kannaiyan, Paola Murgas, Concepción Panadero-Medianero, Jorge Ibañez-Vega, Paula I. Burgos, Eileah Loda, Stephen D. Miller, Moritz J. Rossner, Peter J. Gebicke-Haerter, Rodrigo Naves

**Affiliations:** ^1^ Program of Immunology, Institute of Biomedical Sciences, Faculty of Medicine, Universidad de Chile, Santiago, Chile; ^2^ Department of Microbiology-Immunology, Feinberg School of Medicine, Northwestern University, Chicago, IL, United States; ^3^ Molecular Neurobiology, Department of Psychiatry & Psychotherapy, Ludwig-Maximilians-University of Munich, Munich, Germany; ^4^ Center for Integrative Biology, Faculty of Science, Universidad Mayor, Santiago, Chile; ^5^ Department of Clinical Immunology and Rheumatology , School of Medicine, Pontificia Universidad Católica de Chile, Santiago, Chile; ^6^ Central Institute of Mental Health, Faculty of Medicine, University of Heidelberg, Mannheim, Germany

**Keywords:** multiple sclerosis, experimental autoimmune encephalomyelitis, interferon-gamma, microglia, myeloid cells, neuroinflammation, neurodegenerative disease, immune tolerance.

## Abstract

Compelling evidence has shown that interferon (IFN)-γ has dual effects in multiple sclerosis and in its animal model of experimental autoimmune encephalomyelitis (EAE), with results supporting both a pathogenic and beneficial function. However, the mechanisms whereby IFN-γ may promote neuroprotection in EAE and its effects on central nervous system (CNS)-resident cells have remained an enigma for more than 30 years. In this study, the impact of IFN-γ at the peak of EAE, its effects on CNS infiltrating myeloid cells (MC) and microglia (MG), and the underlying cellular and molecular mechanisms were investigated. IFN-γ administration resulted in disease amelioration and attenuation of neuroinflammation associated with significantly lower frequencies of CNS CD11b^+^ myeloid cells and less infiltration of inflammatory cells and demyelination. A significant reduction in activated MG and enhanced resting MG was determined by flow cytometry and immunohistrochemistry. Primary MC/MG cultures obtained from the spinal cord of IFN-γ-treated EAE mice that were *ex vivo* re-stimulated with a low dose (1 ng/ml) of IFN-γ and neuroantigen, promoted a significantly higher induction of CD4^+^ regulatory T (Treg) cells associated with increased transforming growth factor (TGF)-β secretion. Additionally, IFN-γ-treated primary MC/MG cultures produced significantly lower nitrite in response to LPS challenge than control MC/MG. IFN-γ-treated EAE mice had a significantly higher frequency of CX3CR1^high^ MC/MG and expressed lower levels of program death ligand 1 (PD-L1) than PBS-treated mice. Most CX3CR1^high^PD-L1^low^CD11b^+^Ly6G^-^ cells expressed MG markers (Tmem119, Sall2, and P2ry12), indicating that they represented an enriched MG subset (CX3CR1^high^PD-L1^low^ MG). Amelioration of clinical symptoms and induction of CX3CR1^high^PD-L1^low^ MG by IFN-γ were dependent on STAT-1. RNA-seq analyses revealed that *in vivo* treatment with IFN-γ promoted the induction of homeostatic CX3CR1^high^PD-L1^low^ MG, upregulating the expression of genes associated with tolerogenic and anti-inflammatory roles and down-regulating pro-inflammatory genes. These analyses highlight the master role that IFN-γ plays in regulating microglial activity and provide new insights into the cellular and molecular mechanisms involved in the therapeutic activity of IFN-γ in EAE.

## 1 Introduction

Multiple Sclerosis (MS) is a disease of the central nervous system (CNS) characterized by chronic inflammation and demyelination. It is the most common autoimmune disease in the brain and the leading cause of non-traumatic neurological disability in young adults (1). Experimental autoimmune encephalomyelitis (EAE) remains the animal model most widely used to study disease mechanisms and therapeutic approaches for MS (2). EAE is actively induced by immunization with myelin-derived antigens associated with adjuvant and consists of an induction phase and an effector phase (3). The induction phase involves the priming of myelin epitope-specific CD4^+^ T cells in the periphery. The effector phase is characterized by innate and adaptive immune cell migration from the periphery into the CNS and their re-activation by CNS resident cells such as microglia (MG) or immigrating antigen-presenting cells (APC) (2, 3). Both MS and EAE are characterized by inflammatory lesions in the CNS that mainly contain cells expressing the CD11b cell marker (4). This CD11b^+^ cell population includes peripheral myeloid cells (MC) such as neutrophils, monocytes, dendritic cells, and macrophages as well as CNS resident MG.

MG constitute about 5-20% of all cells in the CNS (5) and their primary role is the support and maintenance of CNS as well as to perform important surveillance functions (6). MG are characterized by a prominent expression of the fractalkine receptor CX3CR1, which is not expressed in astrocytes, oligodendrocytes, or neurons (7, 8). Indeed, CX3CR1 promotor activity has been used for the visualization, genetic manipulation, and the study of the function of MG in the CNS (9). In addition, CX3CR1 is considered a microglial homeostatic marker (10, 11) and lack of this receptor results in exacerbation of inflammation and increased expression of MHC class II molecules in MG (12–14). During early stages of demyelination, active lesions present increased numbers of MG expressing pro-inflammatory markers associated with phagocytosis, antigen presentation and T cell co-stimulation. In later stages, MG develop an intermediate phenotype between pro- and anti-inflammatory activation. Interestingly, loss of homeostatic microglial signature observed in active lesions of MS patients is restored during disease inactivity (15, 16). Therefore, MG have the capability of producing a wide variety of molecules that allow them to exert both inflammatory/detrimental and anti-inflammatory/protective functions in EAE and MS (16).

Interferon gamma (IFN-γ), the only member of the type II IFN family, is a cytokine that has been historically considered the hallmark of Th1 cells driving inflammation in EAE and MS (17, 18). However, compelling evidence has challenged the notion that IFN-γ is strictly pathogenic and has been ascribed a protective role as well [reviewed in (19–21)]. Several studies analyzing EAE development in mice either deficient in the IFN-γ gene, lacking the IFN-γ receptor, or treated with neutralizing antibodies against IFN-γ, demonstrate that endogenous IFN-γ plays a disease-limiting role in EAE (22–33). Likewise, EAE symptoms are ameliorated in response to IFN-γ administered systemically (i.p.) (29, 30) or directly into the CNS (33). Therefore, IFN-γ has opposite effects in EAE, which can be explained, at least in part, through its dose-dependent dual action on MG. Low doses of IFN-γ enable MG to perform neuroprotective functions, whereas high doses of IFN-γ polarize MG toward an inflammatory state [reviewed in (20)]. In EAE, we and other investigators have found that IFN-γ is detrimental during the induction phase but protective during the early effector phase (acute phase) (26, 32, 34, 35), indicating that opposing effects of IFN-γ depend on the stage of the disease. However, the mechanisms whereby IFN-γ is able to exert protection in EAE and its role at the peak of EAE remain unresolved. Moreover, most studies concerning the role of IFN-γ in the pathogenesis and progression of EAE and MS have primarily focused on peripheral lymphoid cells while its action on CNS-infiltrating myeloid cells and CNS-resident cells such as MG has been largely ignored, despite their critical role in regulating autoimmune neuroinflammation. This study aims to elucidate the impact of systemic administration of IFN-γ at the peak of EAE, its effects on CNS infiltrating MC and MG, and the underlying cellular and molecular mechanisms.

## 2 Material and methods

### 2.1 Mice

C57BL/6 mice and the Signal Transducer and Activation Transcription (Stat)-1^-/-^ (B6.129S(Cg)-Stat1tm1Dlv/J, stock #012606) mice were obtained from The Jackson Laboratory. C57BL/6J MOG_35-55_-specific TCR transgenic (2D2) mouse strain was kindly provided by Dr. R Pacheco (Fundación Ciencia & Vida, Chile). All mice were maintained under specific pathogen-free (SPF) conditions. All experimental procedures complied with the Helsinki Declaration of animal experiments and were approved by the Institutional Animal Care and Use Committee (IACUC) of the University of Chile and Northwestern University.

### 2.2 Induction of EAE and treatment

**Figure 1:**
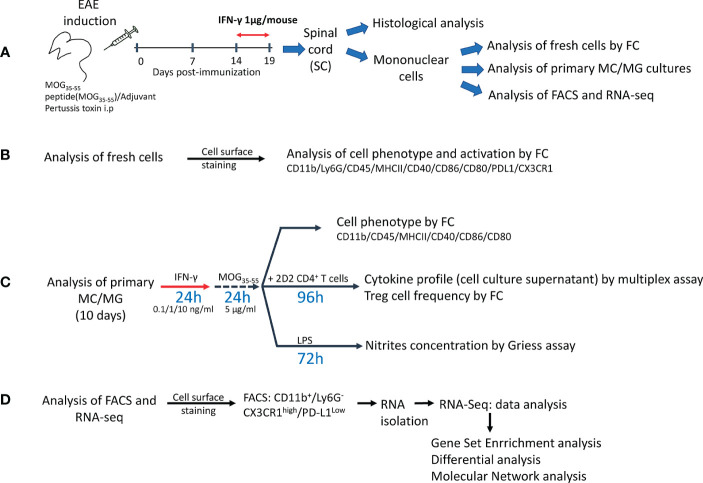
Experimental design. **(A)** Mice were immunized with myelin oligodendrocyte protein peptide (MOG_35-55_) to induce experimental autoimmune encephalomyelitis (EAE). Mice were treated daily with 1 μg/mouse of mrIFN-γ or PBS for 5 days at the peak of EAE. SC were collected for histological analysis or to isolate mononuclear cells, which were used for analysis of fresh cells by multiparametric flow cytometry (FC), primary myeloid cells/microglia (MC/MG) cultures, fluorescence activated cell sorting (FACS), and RNAseq analysis. **(B)** Fresh isolated cells were used to determine the cell phenotype by FC analysis using the markers described in the figure. **(C)** Primary MC/MG cultures were established and then cells were pre-conditioned with low doses of IFN-γ (0.1, 1, and 10 ng/ml) for 24 h and then pulsed with MOG_35-55_ (5 μg/ml) for an additional 24 h. Cells were analyzed for tolerogenic phenotype by FC or were co-cultured with 1x10^6^ CD4^+^ T cells obtained by negative selection from spleens of 2D2 mice. After 4 days of co-culture, supernatants were collected for further cytokine analysis by multiplex assay or ELISA and the cells were analyzed for Treg cell frequency (CD4^+^CD25^high^Foxp3^+^) by FC. In other assays, pre-conditioned MC/MG cultures were challenged with 1 µg/ml LPS for 72 h. Cell culture supernatant was collected and nitrite was determined by Griess reaction. **(D)** Fresh isolated cells were stained with antibodies against CD11b, LY6G, CD45, CX3CR1 and PD-L1, and immediately sorted on a FACSAria™ III. RNA was isolated using RNAeasy Micro kit and used for RNAseq analysis.

EAE was induced in 8- to 12-week-old mice with a s.c. injection of 150 µg myelin oligodendrocyte glycoprotein-derived 35-55 peptide (MOG_35-55_, MEVGWYRSPFSRVVHLYRNGK, CPC Scientific, California, US), emulsified in Incomplete Freund’s adjuvant containing 500 µg *Mycobacterium tuberculosis* (BD Difco, Detroit, Michigan, US) followed by an i.p. injection with 500 ng *Bordetella pertussis* toxin (List Biological, Campbell, California, US) on the day of immunization and 48 h later. Body weight and clinical symptoms were monitored daily using a standard clinical score of 0-6 as previously described (36). For treatment with IFN-γ, 1 μg/mouse/day of recombinant murine IFN-γ (Biolegend, San Diego, California, US) was administered i.p. for 5 days starting at the peak of EAE. Non-immunized (NI) mice (without EAE) and EAE mice injected with phosphate-buffered saline (PBS) (Gibco, Grand Island, New York, US) were used as control groups (**Figure 1A**).

### 2.3 Histological analysis

Mice were deeply anesthetized and intracardially perfused with PBS (0.1 M) followed by 4% paraformaldehyde (pH=7.4). Thoracic and lumbar spinal cord (SC) sections were removed, post-fixed in 4% paraformaldehyde, and embedded in paraffin. Serial sections with 6 μm thickness were cut, followed by hematoxylin and eosin (H&E) and luxol fast blue (LFB) staining. All reagents were purchased from Sigma (Saint Louis, Missouri, USA). LFB images were captured on an Olympus BX51 multichannel light/epifluorescence microscope (Olympus, Tokyo, Japan). H&E images were captured on a NanoZoomer XR slide scanner (Hamamatsu Photonics, Japan) employing the NanoZoomer Digital Pathology scan software v3.0 (Hamamatsu Photonics, Japan). Density of infiltrating inflammatory cells was determined as the number of cell nuclei per 10.000 µm^2^. The extent of demyelination was evaluated by measuring the percentage of demyelinated area over the total white matter area for each SC section. Quantifications were performed using ImageJ software (NIH, USA).

### 2.4 Immunohistochemistry

For immunostaining, thoracic SC sections were deparaffinized and rehydrated. Heat-induced antigen retrieval was performed in citrate buffer (pH=6.0) for 30 min. Sections were washed in 1X Tris-buffered saline (TBS), permeabilized, and blocked for 1 h at room temperature (RT) in blocking buffer [5% bovine serum albumin (BSA), 0.5% Triton-X 100 in 1X TBS)]. Tissues were incubated with the primary antibody polyclonal rabbit anti-ionized-calcium binding adaptor protein (Iba) 1 diluted in blocking buffer (1:300, FujiFilm Wako, Osaka, Japan) overnight at 4°C. The next day, slides were washed with 1X TBS and incubated with Alexa Fluor 555-labeled secondary antibody anti-rabbit (1:200, Invitrogen, Waltham, MA, US) for 3 h at RT (**Table 1**). Slides were washed with 1X TBS, and cell nuclei were stained with 4’,6-diamidino-2-phenylindole (DAPI). Finally, slides were rinsed with 1X TBS, mounted with an anti-fade mounting media, and visualized in a Leica DMI8 inverted fluorescence microscope. Image J-assisted analysis was used to evaluate density of Iba1^+^ cells, determined as the number of Iba1^+^ cells per 100.000 µm^2^; average Iba1^+^ cell size, determined as cell area of Iba1^+^ cells; and percentage Iba1 coverage, determined as the percentage of the total section area occupied by Iba1^+^ cells (µm^2^).

**Table 1:** List of antibodies used for immunofluorescence and flow cytometry.

Specificity	Fluorochrome	Dilution	Source	Clone/ID
Neutrophil				
LY6G	BV605	1:500	BIOLEGEND	1A8
Microglia				
TMEM119	Unconjugated (Rabbit)	1:100	ABCAM	106-6
* *	anti-rabbit Alexa fluor 555	1:1000	INVITROGEN	AB_2535849
Myeloid cells/Microglia				
Iba1	Unconjugated (Rabbit)	1:300	WACO	Polyclonal
	anti-rabbit Alexa fluor 555	1:200	INVITROGEN	AB_2535849
CD11b	FITC/PE	1:300	BIOLEGEND	M1/70
CD45	APC	1:500	BIOLEGEND	30-F11
CX3CR1	PE/Cy7	1:500	BIOLEGEND	SA011F11
PD-L1 (CD274; B7-H1)	BV711	1:400	BIOLEGEND	10F.9G2
MHCII	Alexa Fluor700	1:600	EBIOSCIENCE	MS/114.15.2
CD86	APC/Cy7	1:400	BIOLEGEND	GL-1
CD80	PE	1:200	BIOLEGEND	16-10A1
CD40	PerCP	1:300	BIOLEGEND	3/23
CD4^+^ Treg cells				
CD4	FITC	1:200	BIOLEGEND	RM4-5
CD25	APC	1:200	BIOLEGEND	PC61
FoxpP3	PE	1:200	BIOLEGEND	150D
Myelin				
MBP1	Unconjugated (Mouse)	1:70	BIOLEGEND	SMI99
* *	anti-mouse Alexa fluor 555	1:500	INVITROGEN	AB_2535844

APC, allophycocyanin; BV,brilliant violet; Cy, cyanine; FITC, fluorescein isothiocyanate; PE, phycoerythrin; PERCP, Peridinin Chlorophyll Protein Complex.

### 2.5 Cell cultures and ex vivo re-stimulation

Mononuclear cells were isolated from SC of EAE mice treated with PBS or IFN-γ at day 19 post-immunization as previously described (37). Briefly, spinal cord homogenates were obtained and incubated with 0.5 mg/ml collagenase (Roche, Manheim, Germany) and 10 units/ml DNAse I (New England Biolabs, Ipswich, Massachusetts, US) at 37°C for 1 h. Mononuclear cells were purified using 40%/70% discontinuous Percoll gradients (Amersham, Piscataway, New Jersey, US), and total cell numbers were determined using a hemocytometer with viability assessed by trypan blue exclusion. In some assays, fresh cells were immediately analyzed by flow cytometry (**Figure 1B**). In other experiments, primary cell cultures were established; seeding cells in 24-well plates at a density of 2x10^5^ cells per well in 1 ml Iscove´s Modified Dulbecco´s Medium (IMDM) supplemented with 10% fetal calf serum (FCS), 100U/ml penicillin plus 100 µg/ml streptomycin, 1 mM sodium pyruvate, 50 µM beta-mercaptoethanol, 2 mM glutamine, and non-essential amino acids (all from Gibco, Carlsbad, California, US) (**Figure 1C**). Primary cell cultures usually contained 80-90% of adherent CD11b^+^ myeloid/microglia (MC/MG) (**Supplementary Figure 1**). For nitric oxide (NO) determination, MC/MG were re-stimulated with 1 ng/ml IFN-γ for 24 h and then pulsed with 5 μg/ml MOG_35-55_ for an additional 24 h. Then, re-stimulated MC/MG cultures were challenged with 1 µg/ml bacterial lipopolysaccharide (LPS) for 72 h in IMDM culture media. Thereafter, the medium was collected, and nitrite, a stoichiometric and stable metabolite of NO, was determined from supernatants by Griess reaction (Promega, Madison, Wisconsin, US). For cell co-culture experiments, MC/MG were re-stimulated with varying amounts of IFN-γ (0.1-1-10 ng/ml) for 24 h, washed, and then pulsed with MOG_35-55_ (5 μg/ml) for an additional 24 h. After washing, cells were co-cultured with 1x10^6^ CD4^+^ T cells previously purified by negative selection from spleens of 2D2 mice using a CD4^+^ T cell isolation kit (Miltenyi Biotec, Bergisch Gladbach, Germany). After 4 days of co-culture, cell culture supernatant was collected for further cytokine analysis, and the cells were analyzed for Treg cell frequency (CD4^+^CD25^high^Foxp3^+^) by flow cytometry (**Figure 1C**).

### 2.6 Immune staining, flow cytometry, and FACS Sorting.

For cell surface staining, isolated cells were immediately fixed with fixation buffer (eBioscience, San Diego California, US) overnight at 4°C. For blocking non-specific Fc receptor-mediated antibody binding, cells were incubated with anti-FcγR III/II antibody for 15 min at 4°C in 2% fetal calf serum (FCS) PBS. Then, cells were stained with antibodies against CD11b, lymphocyte antigen 6 complex locus G (LY6G), CD45, CX3C chemokine Receptor 1 (CX3CR1), programmed death ligand 1 (PD-L1), CD86, CD80, CD40, MHC class II (MHC-II) molecules, and transmembrane protein (TMEM) 119 for 30 minutes at 4°C (**Table 1**). For intracellular staining, cells were permeabilized with Permeabilization kit (eBioscience, San Diego California, US) for 30 minutes at RT and then incubated with antibodies against forkhead box P3 (FoxP3) (Biolegend, USA) for 30 minutes at RT. Next, cells were resuspended in PBS and analyzed using a Fortessa Flow Cytometer (BD Biosciences, USA) and FlowJo software (Tree Star, USA). Flow cytometry gating strategies are described in **Supplementary Figure 2.**

For FACS (**Figure 1D**), freshly isolated SC cells of 5-6 mice were incubated with anti-FcγR III/II antibody for 15 min at 4°C in 2% FCS PBS and immune stained with antibodies against CD11b, LY6G, CD45, CX3CR1, PD-L1, for 30 minutes at 4°C. Cells were immediately sorted on a FACSAria™ III (BD Biosciences, US), collected in tubes containing 2.4 ml of RLT lysis buffer (Qiagen, Hilden, Germany), and frozen at -80°C until RNA-Seq analysis. Usually, 6x10^4^ to 1x10^5^ cells were obtained, and cell viability was higher than 97% (not shown). Post sorting analysis confirmed that >97.5% of sorted cells were CD11b^+^LY6G^-^CX3CR1^high^PD-L1^low^.

### 2.7 Cytokine analysis

The concentration of IFN-γ, IL-1β, tumor necrosis factor (TNF)-α, granulocyte macrophage colony-stimulating factor (GM-CSF), IL-2, IL-4, and IL-10 in cell co-culture supernatants was determined by a multiplex assay using Luminex technology (Merck Millipore, Darmstadt, Germany) according to manufacturer´s instructions. Total and active transforming growth factor (TGF)-β production was determined by ELISA (Invitrogen, Vienna, Austria). The standard curve was diluted in medium containing 10% fetal calf serum, so TGF-β production was calculated over the basal level contained in the fetal calf serum (**Figure 1C**).

### 2.8 RNA sequencing analysis

#### 2.8.1 Library preparation and sequencing

RNA was isolated from CD11b^+^Ly6G^-^CX3CR1^high^PD-L1^low^cells obtained from SC of EAE mice treated with either IFN-γ or PBS using RNAeasy Micro kit (Qiagen, Hilden, Germany) (**Figure 1D**). 1 μl of ERCC RNA Spike-In Mix (ThermoFisher, Carlsbad, California, US) diluted 1:5000 was added to the isolated RNA as an external control. cDNA was synthesized using Ovation RNA-Seq System V2 (NuGen, Groningen, Netherlands). 100 ng of cDNA was used as input for fragmentation and followed by library preparation using the IonXpress plus gDNA and Amplicon Library preparation kit (ThermoFisher, Carlsbad, California, US) as described by the manufacturer. The library was then size selected on a 2% E-Gel (ThermoFisher, Carlsbad, California, US). Sample specific barcodes were then added and amplified. Individual sample libraries were quantified using a Kapa Library Quantification Kit (Kapa, Wilmington, Massachusetts, US) using samples diluted 1:200. Equal quantities of individual samples were then pooled and sequenced on an Ion Proton Sequencer.

#### 2.8.2 Data analysis

The raw reads (Fastq) were split into sample-specific reads based on the barcodes. The reads were then checked for sequence quality and sequence repeats. Low-quality bases and short reads were removed from further analysis. The reads were subsequently mapped to the Mus musculus genome (mm10) using TMAP Aligner and quantified using Ensembl annotation 86 using Partek Flow. Genes with a minimum of 5 reads in at least 80% of the samples were considered for further analysis. Differentially expressed genes (DEGs) were determined in R with the DESeq2 package (38). Genes with at least one-fold change and corrected p-Value of less than 0.05 were considered as differentially expressed between IFN-γ-treated and PBS control mice. Gene set enrichment analysis (GSEA) was performed using the gene sets from the Molecular Signatures Database-MsigDB. Overrepresentation analysis was performed using Reactome database (a database of reactions, pathways, and biological processes) (39, 40).

#### 2.8.3 Construction of a molecular network of protein interactions

In order to search for molecular connections between selected genes, each gene was subjected to a nearest neighbor or cluster analysis using the STRING platform (https://string-db.org/). The setting of 100 interactions (custom value) and a minimum score of 0.400 (medium confidence) was chosen. The lists for each gene were entered into “genes.R “ available in “R” (https://cran.r-project.org/bin/windows/base/) and turned into a graphical display by using “igraph”, a network analysis R package (http://igraph.org/r/).

### 2.9 Statistical analysis

Results were analyzed using a Mann–Whitney nonparametric test or one-way ANOVA using GraphPad Prism v.5.0 (GraphPad Software). P values <0.05 were considered statistically significant.

## 3 Results

### 3.1 IFN-γ treatment induces amelioration of the clinical symptoms and attenuation of neuroinflammation at the peak of EAE

**Figure 2:**
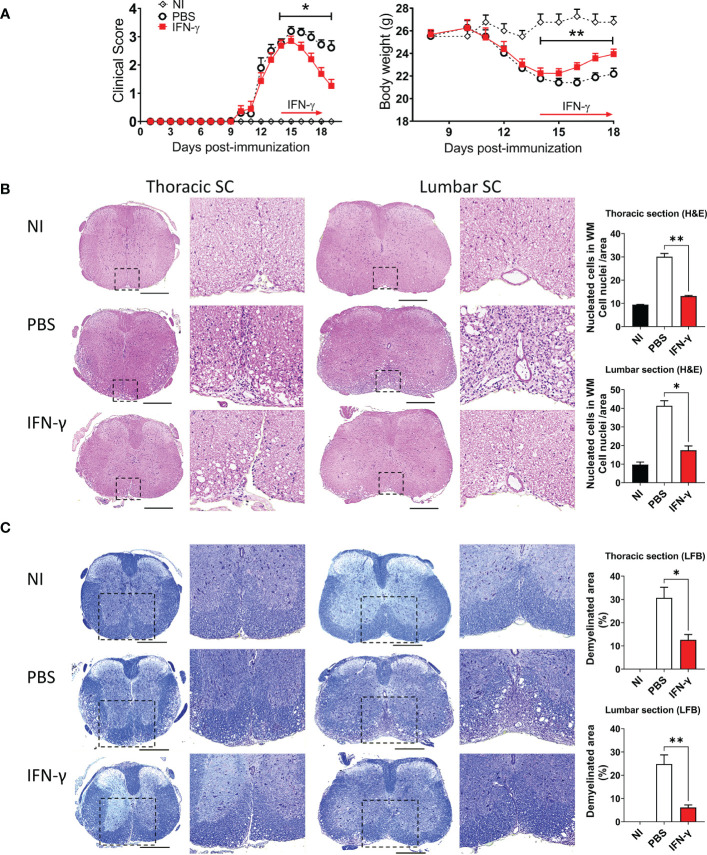
IFN-γ treatment induces disease amelioration and attenuation of neuroinflammation at the peak of EAE. **(A)** Clinical progression and body weight were monitored daily in non-immunized (NI) mice (white diamonds) and mice developing EAE treated with either PBS (black circles) or 1 μg IFN-γ (red squares) for 5 days at the peak of EAE. n= 5 mice per group; 5 independent experiments. **(B, C)** Thoracic and lumbar SC sections from NI, PBS-treated EAE, and IFN-γ-treated EAE mice were analyzed by histochemical staining for **(B)** H&E and **(C)** luxol fast blue. Representative microphotographs are shown. Scale bar is 500 μm. Dashed boxes show magnified image of infiltrated and demyelinated area. Infiltration of inflammatory cells and demyelination area was quantified as described in Methods. All measurements were performed on 3 serial sections per animal (n=5 mice per group). Results are shown as the mean ± SEM. *P <0.05; **P <0.01.

**Figure 3:**
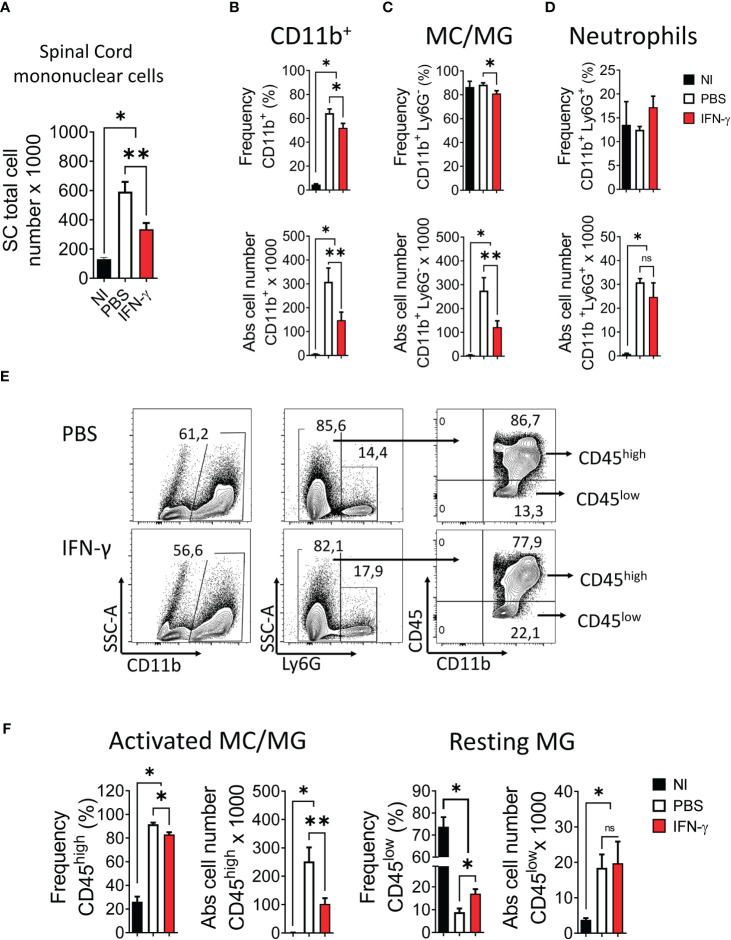
IFN-γ treatment induces a decrease in the number of spinal cord mononuclear cells, a reduction of activated myeloid cell/microglia, and an increase of resting microglia. SC from non-immunized (NI) mice (black bar) and mice developing EAE treated with either PBS (white bar) or 1 μg IFN-γ (red bar) were used to determine **(A)** number of mononuclear cells. **(B-D)** Frequency (top panel) and absolute cell number (bottom panel) of **(B)** CD11b^+^ cells, **(C)** myeloid cell/microglia (MC/MG) (CD11b^+^Ly6G^-^), and **(D)** neutrophils (CD11b^+^Ly6G^+^) were determined by flow cytometry. **(E)** Flow cytometry gating for the determination of activated MC/MG (CD11b^+^Ly6G^-^CD45^high^) and resting MG (CD11b^+^Ly6G^-^CD45^low^). **(F)** Frequency and absolute cell number of activated MC/MG and resting MG were determined by flow cytometry. Values In flow cytometry plots indicate the percentage of positive cells in each gate or quadrant. n= 5 mice per group; 5 independent experiments. Results are shown as the mean ± SEM. *P <0.05; **P <0.01.

First, we determined the effect of systemic administration of IFN-γ for 5 days starting at the peak of EAE. The results showed that IFN-γ significantly decreased the severity of clinical symptoms and body weight loss compared to PBS-treated mice (**Figure 2A**). After cessation of treatment, disease severity returned to levels similar to PBS-treated mice (**Supplementary Figure 3**). Histological analyses showed that thoracic and lumbar SC sections from IFN-γ-treated EAE mice had significantly less infiltration of inflammatory cells and fewer demyelinated areas compared to PBS-treated-EAE mice (**Figures 2B, C**). Interestingly, flow cytometry analysis revealed that *in vivo* IFN-γ-treatment resulted in a significantly lower absolute number of mononuclear cells and lower frequency and absolute numbers of CD11b^+^ cells and non-neutrophil MC/MG (CD11b^+^Ly6G^-^) compared to SC from PBS-treated EAE mice; however, levels were still higher than the non-immunized (NI) group (**Figure 3A–C**). There was no significant difference in the frequency and absolute number of neutrophils (CD11b^+^Ly6G^+^) between the PBS- and IFN-γ-treated mice (**Figure 3D**). Similar total number of cells was determined in draining lymph nodes (dLN) and spleen from IFN-γ-treated EAE mice and control mice. Frequency and absolute number of CD11b^+^ cells and neutrophils and macrophages in dLN was not affected by IFN-γ treatment. In contrast, a significantly lower frequency and absolute number of CD11b^+^ cells and neutrophils were found in spleen from IFN-γ-treated EAE mice compared to those from PBS-treated EAE mice; whilst macrophages were not significantly altered (**Supplementary Figure 4**).

Several studies have reported that in a neuroinflammatory or tumor microenvironment MG are induced to upregulate CD45 expression (5, 41–49). Consequently, an increase in the frequency of CD45^hi^ cells would reflect the activation of CD45^low^ MG into CD45^hi^ cells resembling peripheral infiltrating MC (44–46). Our results showed that IFN-γ-treatment resulted in a significantly lower frequency and absolute number of activated MC/MG cells (CD11b^+^Ly6G^-^CD45^high^) compared to PBS-treatment. In addition, IFN-γ-treatment induced a significant increase in the percentage of resting MG compared to PBS-treated EAE mice; but was still lower than NI mice (**Figure 3E, F**). Consistent with the activation status of these cell populations, activated MC/MG (CD11b^+^Ly6G^-^CD45^high^) obtained from both IFN-γ- and PBS-treated EAE mice showed a significantly higher expression of MHC-II molecules, CD80, CD40, and PD-L1 than resting MG (CD11b^+^Ly6G^-^CD45^low^) (**Supplementary Figure 5**).

**Figure 4:**
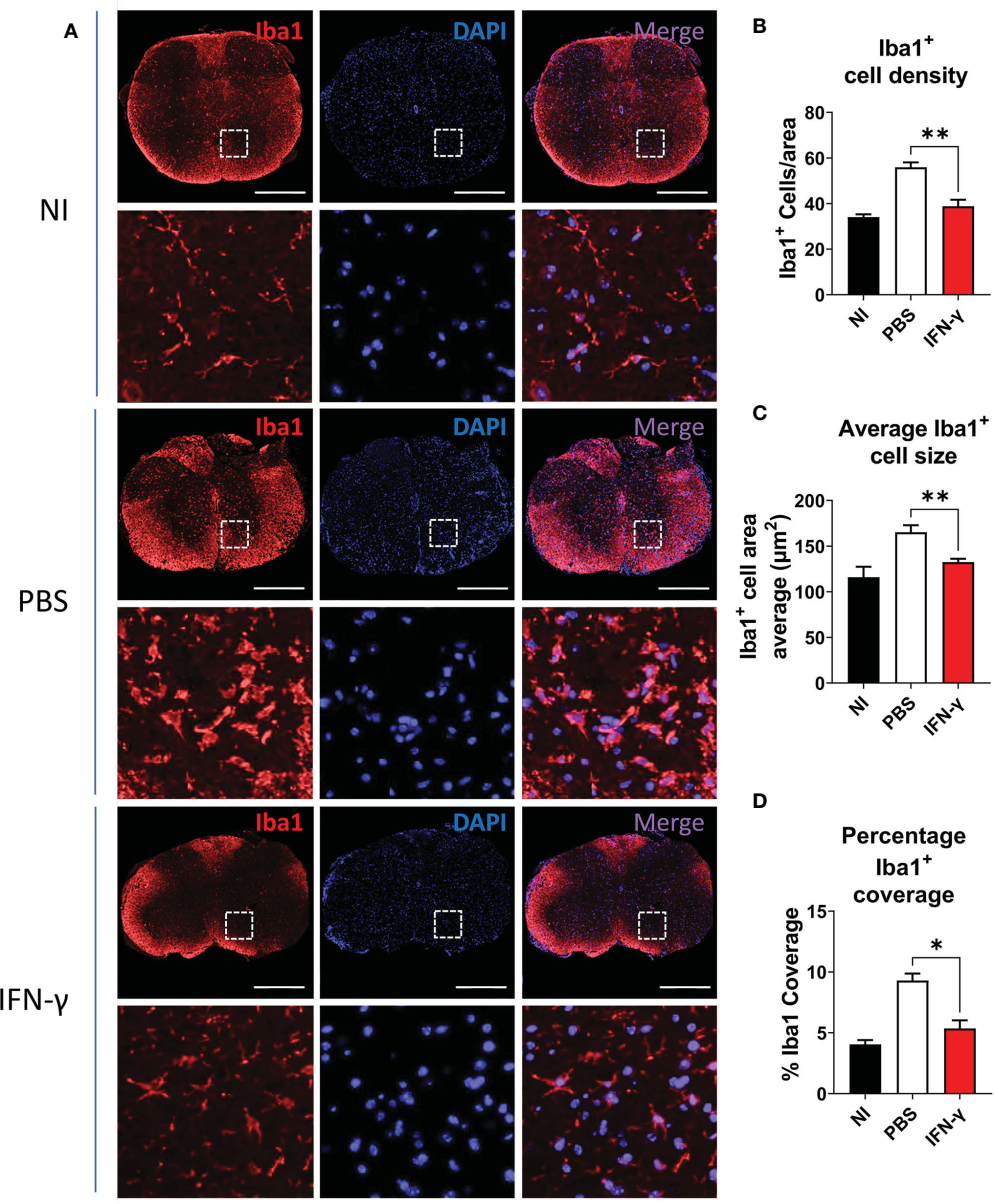
IFN-γ treatment reduces myeloid cell/microglia activation in EAE mice. **(A)** Representative microphotographs of thoracic SC sections from non-immunized (NI) mice, PBS-treated EAE mice (PBS), and IFN-γ-treated EAE mice (IFN-γ) immunostained for Iba1 (red). Cell nuclei were labeled with DAPI (blue). **(B–D)** Determination of **(B)** density of Iba1^+^ cells (number of Iba1^+^ cells per area), **(C)** average Iba1^+^ cell area, and **(D)** percentage Iba1 coverage (percentage of the total section area occupied by Iba1^+^ cells). All measurements were performed on 3 serial sections per animal (n=5 mice per group). Scale bar is 500 μm. Results are shown as the mean ± SEM. *P <0.05; **P <0.01.

Resting MG are also characterized by a ramified cell morphology with numerous thin processes that upon activation are drawn back into the soma, resulting in a rounded amoeboid-like appearance. In order to evaluate morphological changes associated with microglial activation, thoracic SC sections from NI mice and IFN-γ- and PBS-treated EAE mice were immunostained for Iba1, a known cell marker used to evaluate MC/MG morphology and activation (50–54). SC from PBS-treated mice were characterized by extensive Iba1 staining and amoeboid-shaped Iba1^+^ cells. Instead, IFN-γ-treated mice had predominantly ramified-shaped, Iba1-stained cells similar to NI mice (**Figure 4A**). In addition, SC from IFN-γ-treated EAE mice showed the density of Iba1^+^ cells, average Iba1^+^ cell area (indicative of cell size), and percentage Iba1 coverage (indicative of simultaneous alterations in cell density and morphology) significantly reduced in comparison to PBS-treated mice, but similar to NI mice (**Figure 4B–D**).

IFN-γ also plays a protective role in preventing hindbrain neuroinflammation, an effect dependent on the interaction between IFN-γ and CNS cells (55–57). Therefore, changes in MG and myelination in the cerebellum obtained from EAE mice treated with IFN-γ or PBS by immunofluorescence were examined. A significantly higher expression of TMEM119, a recently described homeostatic marker for MG (11, 58), and of MBP staining was observed in response to IFN-γ compared to control treatment (**Supplementary Figure 6**; Method S1). Taken together, these results indicate that *in vivo* IFN-γ-treatment starting at the peak of EAE induces amelioration of clinical symptoms, reduction of body weight loss, attenuation of neuroinflammation associated with significantly less infiltration of inflammatory cells and demyelination, reduced activation of MC/MG, and enhanced expression of homeostatic microglial markers.

### 3.2 Ex vivo re-stimulation with low doses of IFN-γ and MOG_35-55_ induces tolerogenic and anti-inflammatory activity in MC/MG from IFN-γ-treated EAE mice

**Figure 5:**
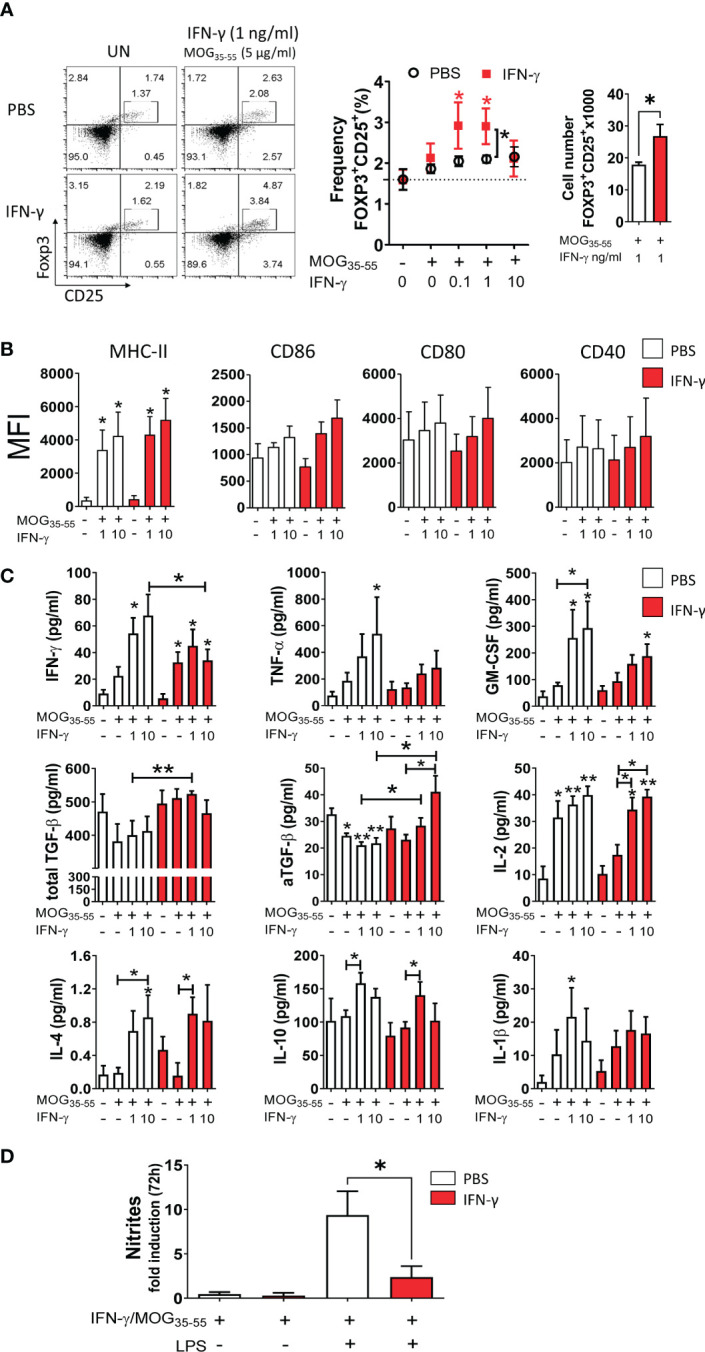
*Ex vivo* re-stimulation with low doses of IFN-γ and MOG_35-55_ induces tolerogenic activity in myeloid cell/microglia from IFN-γ-treated EAE mice. **(A)** Primary MC/MG culture from EAE mice treated with IFN-γ (red squares) or PBS (white circles) were *ex vivo* pre-conditioned with low concentrations of IFN-γ (0.1, 1, and 10 ng/ml) for 24 h, incubated with 5 μg/ml MOG_35-55_ for an additional 24 h, and then co-cultured with purified CD4^+^ T cells obtained from spleens of transgenic 2D2 mice for 96 h. The frequency of Treg cells (CD4^+^CD25^high^FoxP3^+^) was determined by flow cytometry. Representative flow cytometry plots and frequency and cell number of Tregs in co-cultures containing untreated (UN) or pre-conditioned MC/MG with 1 ng/ml IFN-γ and 5 μg/ml MOG_35-55_ is shown. **(B)** The cell surface expression, shown as median fluorescence intensity (MFI), of MHC-II molecules, CD86, CD80, and CD40 was determined by flow cytometry in primary MC/MG obtained from EAE mice treated with IFN-γ (red bars) or PBS (white bars) and pre-conditioned with 1 or 10 ng/ml IFN-γ and 5 μg/ml MOG_35-55_ prior to culture with 2D2 CD4^+^ T cells. **(C)** Secretion of IFN-γ, TNF-α, GM-CSF, IL-2, IL-4, IL-10, and IL-1β was determined by multiplex assay and the production of TGF-β by ELISA in cell culture supernatants from co-cultures between 2D2 CD4^+^ T cells and pre-conditioned MC/MG. **(D)** Production of nitrites was determined by Griess assay in primary MC/MG obtained from EAE mice treated with IFN-γ (red bars) or PBS (white bars) pre-conditioned with 1 ng/ml IFN-γ and 5 μg/ml MOG_35-55_ (IFN-γ/MOG_35-55_) and challenged with 1 μg/ml LPS for 72 h. Results are shown as mean ± SEM of five independent experiments. *Comparison between unstimulated cell cultures and cell cultures stimulated with IFN-γ. Other relevant comparisons are shown with brackets. *P <0.05; **P <0.01. aTGF-β: active transforming growth factor.

It has been reported that low concentrations of IFN-γ induce a tolerogenic phenotype in MG from neonatal mice capable of inducing regulatory T (Treg) cells (59). Thus, we were interested in determining the tolerogenic activity and phenotype of primary MC/MG cultures established from EAE-induced mice treated with IFN-γ or PBS. First, cell cultures were *ex vivo* pre-conditioned with low doses (0.1, 1, and 10 ng/ml) of IFN-γ and MOG_35-55_, and then co-cultured with CD4^+^ T cells obtained from transgenic 2D2 mice for 96 h (**Figure 1C**). The results showed that MC/MG obtained from IFN-γ-treated EAE mice and *ex vivo* stimulated with 0.1 and 1 ng/ml IFN-γ induced a significantly higher frequency of Treg cells compared to untreated cells. Interestingly, *ex vivo* re-stimulation with 1 ng/ml IFN-γ and MOG_35-55_ induced a significantly higher frequency and absolute number of Treg cells in co-cultures containing MC/MG obtained from IFN-γ-treated EAE mice than in co-cultures containing MC/MG from PBS-treated EAE mice (**Figure 5A**). No differences in the expression of MHC-II, CD86, CD80, and CD40 in primary MC/MG obtained from either IFN-γ-treated EAE mice or PBS-treated mice and stimulated with low doses of IFN-γ and MOG_35-55_ were detected prior to culture with 2D2 CD4^+^ T cells (**Figure 5B**). Cell culture supernatants obtained at the end of the co-cultures of pre-conditioned MC/MG and 2D2 CD4^+^ T cells were analyzed by immunoassays. Cell co-cultures containing MC/MG obtained from IFN-γ-treated EAE mice had lower production of IFN-γ, TNF-α, and GM-CSF than conditioned cell co-cultures containing MC/MG obtained from PBS-treated EAE mice. However, only the secretion of IFN-γ in co-cultures containing MC/MG from IFN-γ-treated EAE mice and pre-conditioned with 10 ng/ml IFN-γ and MOG_35-55_ was statistically lower than in those from control mice. Interestingly, cell co-cultures containing MC/MG isolated from IFN-γ-treated EAE mice and *ex vivo* pre-conditioned with 1 ng/ml IFN-γ and MOG_35-55_ showed a significantly higher production of total TGF-β than control co-cultures (**Figure 5C**). Furthermore, active TGF-β production was higher in co-cultures containing MC/MG isolated from IFN-γ-treated EAE mice and *ex vivo* conditioned with 1 and 10 ng/ml IFN-γ and MOG_35-55_ than control co-cultures (**Figure 5C**). In contrast, cell co-cultures containing MC/MG obtained from PBS-treated EAE mice and stimulated *ex vivo* with low doses of MOG_35-55_ alone or in combination with IFN-γ produced significantly lower levels of active TGF-β than unstimulated cell co-cultures. There was no difference in the production of IL-4, IL-10, IL-2, and IL-1β between both groups of co-cultures. These results suggest that *ex vivo* re-stimulation with low doses of IFN-γ and MOG_35-55_ endow MC/MG with the capacity to induce conversion of CD4^+^ T cells into Treg cells in association with high secretion of TGF-β.

Since nitric oxide (NO) is recognized as an important effector molecule produced by macrophages and microglia in response to inflammation (60), we evaluated NO production. Pre-conditioned MC/MG cultures obtained from IFN-γ-treated EAE mice produced significantly lower nitrite in response to LPS stimulation than those from PBS-treated EAE mice (**Figure 5D**). Taken together, these results show that *ex vivo* re-stimulation with low doses of IFN-γ and MOG_35-55_ induces tolerogenic and anti-inflammatory activity in MC/MG.

### 3.3 In vivo IFN-γ-treatment induces increased frequency of CX3CR1^high^PD-L1^low^ MG in a STAT-1-dependent manner

**Figure 6:**
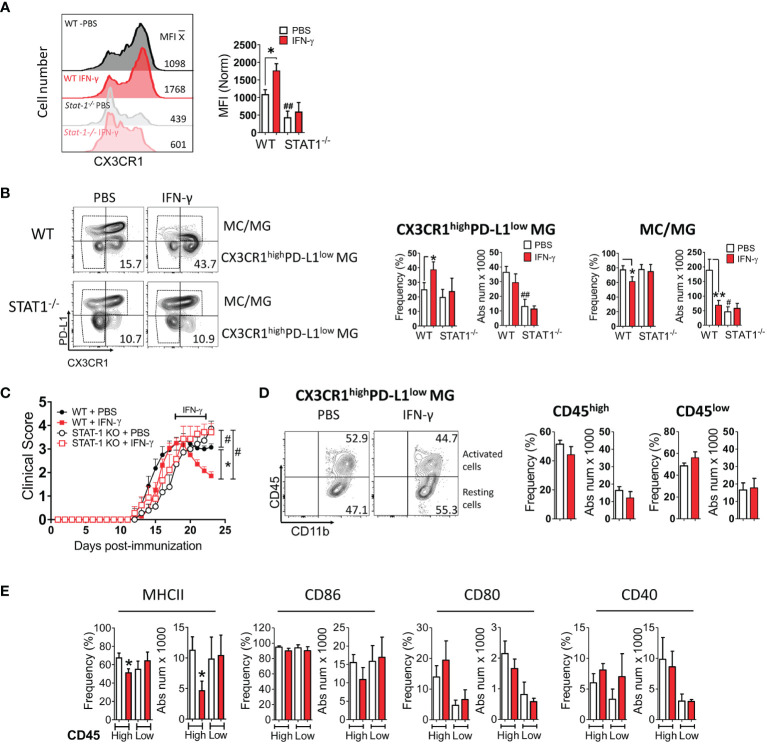
*In vivo* IFN-γ-treatment induces increased frequency of CX3CR1^high^PD-L1^low^ MG in a STAT-1-dependent manner. Mononuclear cells from SC of EAE WT and STAT-1^-^/^-^ mice treated with IFN-γ or PBS for 5 days at the peak of EAE were analyzed by flow cytometry. **(A)** The expression level of CX3CR1. **(B)** Frequency and absolute cell number of CX3CR1^high^ PD-L1^low^ MG and MC/MG in WT and STAT-1^-^/^-^ mice treated with IFN-γ or PBS. **(C)** EAE progression in WT (filled symbols) and STAT-1^-^/^-^ (empty symbols) mice treated with IFN-γ (red line) or PBS (black line) for 5 days at the peak of EAE. **(D)** Frequency and absolute cell number of activated (CD11b^+^CD45^high^) and resting (CD11b^+^CD45^low^) CX3CR1^high^PD-L1^low^ MG and **(E)** their expression of MHC-II, CD86, CD80, and CD40 molecules. *Comparison between mice or cells obtained from IFN-γ-treated EAE mice and control cells obtained from PBS-treated EAE mice. *P <0.05; **P <0.01. ^#^Comparison between mice or cells obtained from WT and STAT1^-/-^ mice. ^#^P <0.05; ^##^P <0.01. MFI, median fluorescence intensity.

Next, we examined the expression of MHC class II molecules, costimulatory molecules (CD80, CD86, CD40), coinhibitory molecules (PD-L1), and a microglial marker (CX3CR1) in MC/MG obtained from IFN-γ and PBS-treated EAE mice by flow cytometry (**Supplementary Figure 7A**). Interestingly, we found that *in vivo* IFN-γ treatment induced a significantly higher expression of CX3CR1 in MC/MG cells than in control cells (**Figure 6A**). In turn, an analysis of CX3CR1^high^CD11b^+^Ly6G^-^ cells showed that IFN-γ treatment induced a significantly higher frequency of these cells expressing low PD-L1 (**Figure 6B**; **Supplementary Figure 7B**). There was no difference in the absolute number of CX3CR1^high^PD-L1^low^CD11b^+^Ly6G^-^ cells from mice treated with IFN-γ or PBS. However, the total number of live mononuclear cells isolated from the SC of IFN-γ-treated EAE mice was almost half of PBS-treated EAE mice (**Figure 3A**). These CX3CR1^high^PD-L1^low^CD11b^+^Ly6G^-^ cells were 75-85% TMEM119^+^ (**Supplementary Figure 8A**). In addition, CX3CR1^high^PD-L1^low^CD11b^+^Ly6G^-^ cells showed a strong expression of gene markers for MG and weak expression for MC, oligodendrocytes, and astrocytes (**Supplementary Figure 8B**). Therefore, these results strongly suggest that this cell subpopulation is an enriched subset of CX3CR1^high^ MG expressing low PD-L1 (CX3CR1^high^PD-L1^low^ MG). The remaining cells were 47-52% TMEM119^+^ (**Supplementary Figure 8A**) and were considered MC/MG. A significantly lower frequency and absolute number of MC/MG was observed in SC from IFN-γ-treated EAE mice than in SC from PBS-treated EAE mice (**Figure 6B**). Interestingly, NI mice exhibited a significantly higher frequency and absolute number of CX3CR1^high^PD-L1^low^ MG (60.2% ± 5.4%) than MC/MG (39.5% ± 5.6%) (**Supplementary Figure 9A**).

To determine whether the increase in CX3CR1^high^PD-L1^low^ MG induced by IFN-γ is due to proliferation, mice treated with IFN-γ or PBS received a simultaneous i.p. injection of 5-bromo-2´-deoxyuridine (BrdU) starting at the peak of EAE. After 5 days, mononuclear cells were isolated from SC, and the frequency of proliferating CX3CR1^high^PD-L1^low^ MG was analyzed by flow cytometry. The results showed that there was no difference in the frequency of proliferating CX3CR1^high^PD-L1^low^ MG between EAE mice treated with IFN-γ or PBS, suggesting that enhanced CX3CR1^high^PD-L1^low^ MG induced by IFN-γ might be explained by microglial plasticity (**Supplementary Figure 9**; Method S2).

To obtain mechanistic insight into the induction of CX3CR1^high^PD-L1^low^ MG by IFN-γ, mice lacking STAT-1, the major STAT activated in response to engagement of IFN-γ receptor, were induced with EAE and then treated with IFN-γ for 5 days starting at the peak of disease. IFN-γ treatment had no effect on disease progression in EAE-induced *Stat*-1^-^/^-^ mice (**Figure 6C**). Furthermore, the lack of STAT-1 inhibited the IFN-γ-induced expression of CX3CR1 in MC/MG cells (**Figure 6A**), suppressed the increase of CX3CR1^high^PD-L1^low^ MG, and reversed the decreased frequency of MC/MG induced by IFN-γ in WT mice (**Figure 6B**). We thus conclude that the IFN-γ/STAT-1 signaling axis is involved in symptom amelioration and induction of CX3CR1^high^PD-L1^low^ MG in EAE.

Next, we analyzed the impact of IFN-γ-treatment on the activation state and the expression of MHC-II molecules and costimulatory molecules in CX3CR1^high^PD-L1^low^ MG from EAE-induced WT mice treated with IFN-γ or PBS. A similar frequency of activated (51.5 ± 6%) and resting (44.2 ± 12%) CX3CR1^high^PD-L1^low^ MG was found in SC from IFN-γ- and PBS-treated EAE mice (**Figure 6D**). However, activated CX3CR1^high^PD-L1^low^ MG from IFN-γ-treated EAE mice exhibited a significantly lower frequency and absolute number of cells expressing MHC-II molecules than cells from PBS-treated EAE mice (**Figure 6E**). There was no significant difference in the frequency or absolute number of cells expressing CD80, CD86, or CD40 between CX3CR1^high^PD-L1^low^ MG from IFN-γ- and PBS-treated EAE mice. Interestingly, in NI mice most of CX3CR1^high^PD-L1^low^ MG (91.9% ± 4.2%) were in a resting state and had significantly lower frequency of cells expressing CD40 compared to activated cells, whereas the expression of MHC-II molecules, CD86, and CD80 were not statistically significant (**Supplementary Figure 9B**).

### 3.4 Differential gene expression profile of MG isolated from IFN-γ or PBS-treated EAE mice

**Figure 7:**
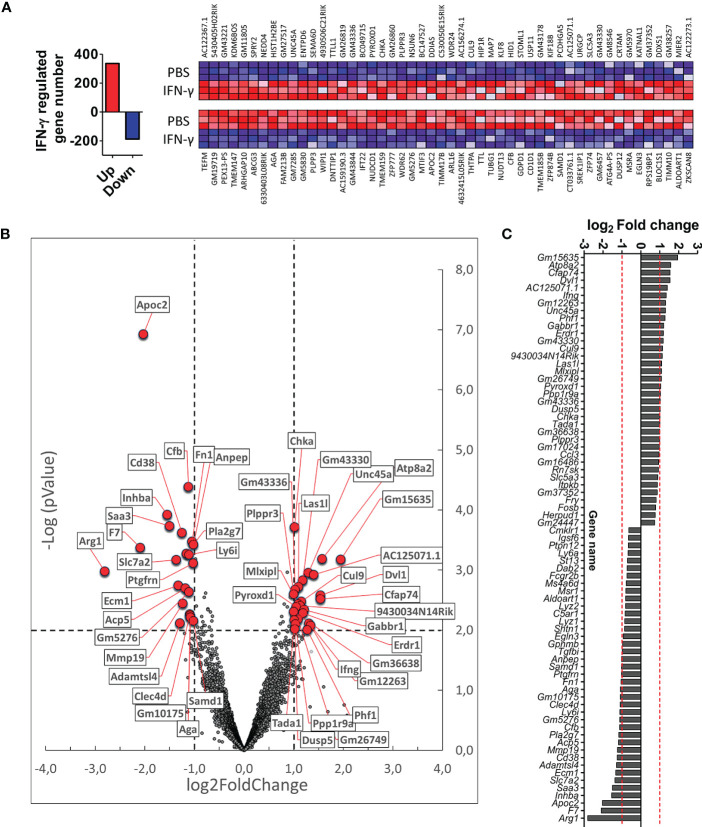
Differential gene expression profile in CX3CR1^high^PD-L1^low^ MG in response to *in vivo* IFN-γ-treatment. Gene expression profile was analyzed on RNA obtained from CX3CR1^high^ MG expressing low PD-L1 purified from SC of IFN-γ or PBS-treated EAE mice by RNA sequencing (RNA-seq). **(A)** Left panel, total number of genes up (red bar)- and down (blue bar)-regulated by IFN-γ treatment; right panel, heat map showing the top 100 up (red squares)- and down (blue squares)-regulated genes in CX3CR1^high^PD-L1^low^ MG from IFN-γ-treated mice versus those cells from PBS-treated mice. **(B)** Volcano plot showing up- and down-regulated genes induced by IFN-γ in CX3CR1^high^PD-L1^low^ MG from IFN-γ-treated mice. **(C)** Gene expression analysis using more stringent criteria by lowering the p-value to 0.01, setting the Log2 Fold change to a minimum of 1.0, and by raising the threshold of minimum expression to 40 units. n= 3 independent samples per group.

We next wanted to identify genes and signaling pathways in which IFN-γ may be differentially regulating the activity of CX3CR1^high^PD-L1^low^ MG in EAE by analyzing the transcriptional profile of these cells. CX3CR1^high^PD-L1^low^ MG were isolated from IFN-γ- and PBS-treated EAE mice for FACS sorter (97.5% purity), and the transcriptional profile was analyzed by RNAseq. A total of 12,524 genes were detected above the threshold (see Methods), with 336 genes upregulated and 188 down-regulated after IFN-γ treatment (**Figure 7A**). Using more stringent criteria, lowering the p-value to 0.01, setting the Log2 Fold change to a minimum of 1.0, and raising the threshold of minimum expression to 40 read units, resulted in 25 upregulated and 22 down-regulated genes in response to IFN-γ (**Figures 7B, C**). Interestingly, the gene expression of *ATPase phospholipid transporting 8A2* (*Atp8a2*), a selective microglial gene marker, and several genes associated with anti-inflammatory processes such as *dual-specificity phosphatase 5* (*Dusp5*), *MLX interacting protein-like* (*Mlxipl*, *ChREBP*), *dishevelled segment polarity protein 1* (*Dvl1*) and *gamma-aminobutyric acid type B receptor subunit 1* (*Gabbr1*), were upregulated in IFN-γ-treated EAE CX3CR1^high^PD-L1^low^ MG (**Figures 7B, C**; **Table 2**). Several genes associated with M1 inflammatory activity such as the *cd38 molecule* (*Cd38*)*, complement factor B* (*Cfb*)*, inhibin subunit Beta A* (*Inhba*)*, serum amyloid A3* (*Saa3*), *and* APN-like peptidase cytosolic alanyl-aminopeptidase *(Anpep)* were down-regulated in IFN-γ-treated EAE CX3CR1^high^PD-L1^low^ MG (**Figures 7B, C**; **Table 2**). Unexpectedly, *arginase 1* (*Arg1*), a M2 classic gene, was down-regulated in CX3CR1^high^PD-L1^low^ MG from IFN-γ-treated EAE mice. However, the cationic amino acid transporter 2 (*Slc7a2*), a gene involved in the uptake of arginine, was also down-regulated, suggesting a decreased substrate availability for NO production in these cells (**Figures 7B, C**; **Table 2**).

**Table 2:** Role of genes regulated by IFN-γ.

Up	Name	Activity	Role	Ref
*Atp8a2*	ATPase Phospholipid Transporting 8A2	Lipid flipping: generating and maintaining asymmetry in membrane lipid	Selective microglial marker	(117)
*Dusp5*	Dual specificity phosphatase 5	Mitogen-activated protein kinase phosphatase	Inhibits production of TNF-α and IL-6 by inactivating ERK 1/2 pathway	(61, 62)
*Mixipl (ChREBP)*	Carbohydrate-responsive element binding protein	Transcription factor involved in regulation and maintenance of macrophages redox status	Prevents macrophage inflammatory responses	(63)
*Dvl1*	Dishevelled Segment Polarity Protein 1	Cytoplasmic phosphoprotein	Participates in the Wnt/β-catenin signaling pathway inducing tolerogenic DC	(64, 65)
*Gabbr1*	Gamma-Aminobutyric Acid Type B Receptor Subunit 1	Metabotropic GABAB inhibitory G-coupled receptor	Inhibits LPS-induced IL-6 and IL-12p40 expression in microglia	(66, 67)
*Ifn-y*	Interferon-y	Cytokine	Pro- and anti-inflammatory role	(19)
Down	Name	Activity	Role	Ref
*Cd38*	CD38	Transmembrane enzyme that synthesizes and hydrolyzes cADP-ribose	Associated to M1 macrophages activity inducing activation of iNOS and production of TNF-α, IL-6 and IL-1β. EAE mice lacking CD38 showed ameliorated disease.	(68–71)
*Cfb*	Complement Factor B	Component of the alternative pathway complement activation	Associated to M1 macrophages activity. Enhanced expression of Cfb in microglia is associated with late stages of neurodegeneration. Inhibition of alternative complement pathway in EAE attenuated the chronic phase of disease.	(72)
*Inhba*	Inhibin Subunit Beta A (Activin A)	Member of TGF-β- superfamily proteins	Induces M1 macrophage polarization and is considered a canonical M1 marker. Blocking anti-Activin A antibody reduced M1 macrophage polarization	(73, 74)
*Saa3*	Serum amyloid A3	Acute phase lipoprotein	Associated to M1 macrophage activity. Considered a pro-inflammatory biomarker involved in releasing active IL-1β by the activation of the NLRP3 inflammasome in LPS-induced microglia	(75–77)
*Anpep*	Alanyl Aminopeptidase, membrane (CD13)	aminopeptidase	Involved in adhesion of monocytes to endothelial cells and trafficking toward inflammation. Pharmacological inhibition of ANPEP induced amelioration of EAE.	(110–111)
*Slc7a2*	Solute Carrier Family 7 Member 2 (CAT2)	Cationic amino acid transporter	Responsible for the cellular uptake of arginine, lysine, and ornithine. Controls critical aspects of macrophage activation	(78)
*Arg 1*	Arginase 1	Catalyzes the hydrolysis of arginine to ornithine	A canonical M2 marker	(73)

### 3.5 IFN-γ-treatment induces an anti-inflammatory profile in MG

**Figure 8:**
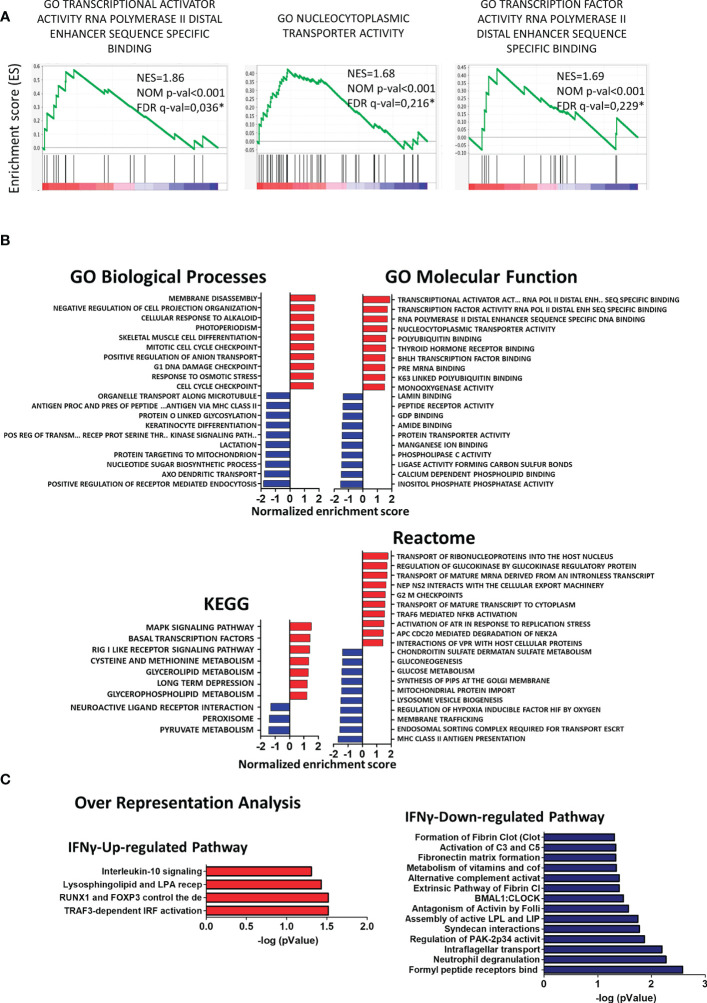
Gene set enrichment analysis (GSEA) and over-representation analysis in CX3CR1^high^PD-L1^low^ MG in response to *in vivo* IFN-γ-treatment. GSEA enrichment score plots show **(A)** up-regulation of gene sets corresponding to RNA transcription and to nucleus-cytoplasmatic transporter activity in CX3CR1^high^PD-L1^low^ MG from IFN-γ-treated EAE mice compared to those cells from PBS-treated EAE mice. Each bar at the bottom of each panel represents a member gene of the respective pathway and shows its relative location in the ranked list. **(B)** Normalized enrichment scores indicate the distribution of Gene Ontology categories across a list of genes ranked by hypergeometrical score (HGS). Higher enrichment scores indicate a shift of genes belonging to certain GO, KEGG, or Reactome categories toward either end of the ranked list, representing up or down-regulation (positive or negative values, respectively). **(C)** Over-representation analysis showing significantly up- and down-regulated cellular pathways in MG from IFN-γ-treated EAE mice compared to MG from PBS-treated EAE mice. n=3 independent samples per group.

To determine if any molecular pathways were differentially regulated in CX3CR1^high^PD-L1^low^ MG by IFN-γ, changes in the transcriptional profile were analyzed by gene set enrichment analysis (GSEA) using different reference databases. Using Gene Ontology (GO) biological processes and Reactome databases, we found that gene sets corresponding to RNA transcription and nucleus-cytoplasmatic transporter activity were highly and significantly upregulated in CX3CR1^high^PD-L1^low^ MG from IFN-γ-treated EAE mice, compared to the control group (*p*< 0.001); indicating enhanced transcriptional and translational activities in response to IFN-γ stimulation (**Figure 8A**). The Kyoto Encyclopedia of Genes and Genomes (KEGG) analysis showed that the top enriched gene set induced by IFN-γ were related to signaling transduction pathways (MAPK and RIG I like receptor), transcription factors (Basal transcription factors), and metabolism (Methionine and Glycerolipids metabolism), which might be related to a change in the activation pattern, oxidative status and lipid-related metabolism associated with a decreased inflammatory profile. Instead, downregulated enriched gene sets in CX3CR1^high^PD-L1^low^ MG from IFN-γ-treated EAE mice were associated with oxidative metabolism (pyruvate metabolism), lipid metabolism, nitric oxide induction (peroxisome), and G-protein coupled receptor-associated to a neuroantigen response (neuroactive ligand-receptor interaction) (**Figure 8B**). Remarkably, over-representation analysis using the Reactome database showed that IFN-γ significantly induced an anti-inflammatory profile in EAE MG. Regarding immune cell function, genes in the TRAF3-dependent IRF activation pathway and Interleukin-10 signaling pathway were upregulated, while neutrophil degranulation, alternative complement activation, and activation of C3 and C5 pathways were down-regulated in CX3CR1^high^PD-L1^low^ MG from IFN-γ-treated EAE mice (**Figure 8C**). Taken together, these results confirm that IFN-γ is a key inducer of anti-inflammatory pathways and suppressor of inflammatory mechanisms in EAE MG.

Because our results indicate the importance of IFN-γ/STAT-1 axis in the induction of CX3CR1^high^PD-L1^low^ MG in EAE, STAT-1 target genes were analyzed in our RNAseq database using the Harmonizome database (p< 0.05) (79). The analysis revealed that 8 genes (*Cfb, Dusp5, Anxa4, Neurl1b, C3, Naca, Anxa11, Slc15a2*) were regulated by STAT-1. Using more stringent criteria (p< 0.01), Cfb and Dusp5 were functionally associated with STAT-1.

### 3.6 IFN-γ establishes tight connections with clusters of down-regulated inflammatory genes and with upregulated anti-inflammatory genes

**Figure 9:**
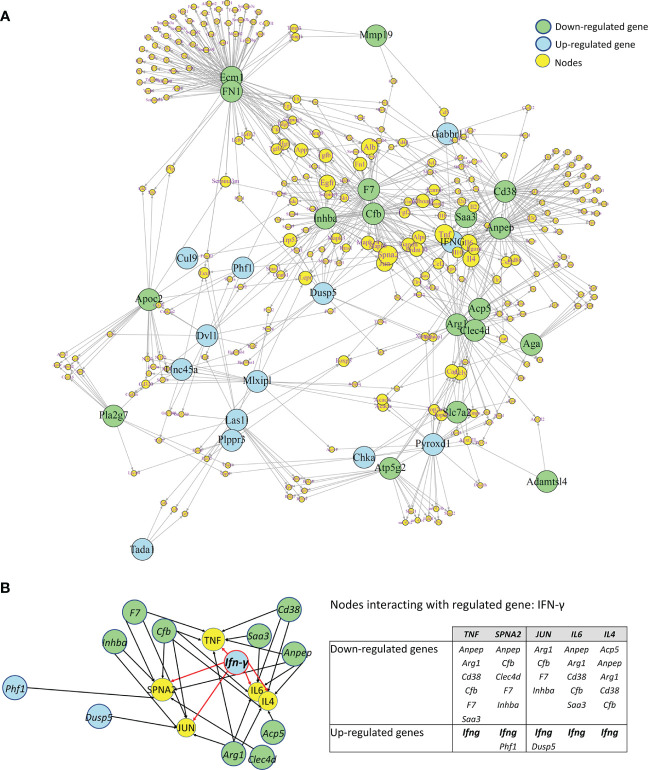
Molecular Network of differentially regulated genes by IFN-γ in CX3CR1^high^PD-L1^low^ MG. **(A)** A molecular network of protein-protein interactions based on the known interactions between the gene products of 31 regulated genes regulated by IFN-γ in an enriched subset of CX3CR1^high^PD-L1^low^ MG. Blue and green circles show down- and upregulated genes by IFN-γ, respectively. Yellow circles represent secondary nodes (connector genes), and their size represents the number of connections with regulated genes. Lines indicate the interaction of regulated genes with connector genes. **(B)** An enlarged area of the molecular network showing that *Ifn-γ* associated with connectors (yellow circles) interacting with three clusters of down-regulated inflammatory genes (green circles) as well as with connectors interacting with upregulated genes (blue circles) associated with anti-inflammatory roles. The table summarizes the up- and down-regulated genes by IFN-γ that interact with each connector.

In order to search for molecular connections among genes regulated by IFN-γ-treatment, a molecular network of protein-protein interactions has been constructed based on the known interactions between the gene products of the 25 up- and 22 down-regulated genes by IFN-γ using the STRING database. Some regulated genes could not be considered because no interacting proteins were found in the STRING database. This resulted in 31 regulated gene products (primary nodes), 13 up-regulated, and 18 down-regulated by IFN-γ-treatment, with various numbers – up to 100 – of interacting proteins (secondary nodes) with each primary node. All secondary nodes were then searched for their occurrence in columns of at least two regulated gene products (**Figure 9A**). Inserting edges (connections) of all primary nodes with secondary nodes identified in their respective columns gave rise to the molecular network displayed in **Figure 9**. As expected, *Ifn-γ* interacted with secondary nodes associated with inflammation, such as TNF-α, IL-6, IL-4, TGF-β1, or IL-10 (**Figure 9A**). Interestingly, we also found that *Ifn-γ* associated with secondary nodes interacting with three clusters of down-regulated inflammatory genes (cluster 1: *Inhba, Cfb, and F7; cluster 2: Saa3, Cd38, and Anpep; cluster 3: acid phosphatase 5, tartrate resistant* (*Acp5*)*, c-type lectin domain family 4 member d* (*Clec4d*), *and Arg1*) and with secondary nodes interacting with up-regulated genes associated with anti-inflammatory roles (*Dusp5, Mlxipl, and Gabbr1*) (**Figures 9A, B**; **Table 2**).

## 4 Discussion

The role of IFN-γ in EAE and MS is still controversial, with evidence supporting both a pathogenic and beneficial function. Some studies have suggested that IFN-γ may have dual activity in these diseases depending on the dose, target cell, and stage of the disease [reviewed in (19–21)]. However, the neuroprotective mechanisms of IFN-γ in EAE remain largely unclear. It is shown here, for the first time, that IFN-γ has therapeutic activity at the peak of EAE by suppressing neuroinflammation and inducing tolerogenic activity of MC/MG and STAT-1-dependent homeostatic adaptation of MG.

Our results showed that IFN-γ-treatment resulted in a significant amelioration of clinical symptoms and reduction of body weight loss. Consistently, SC from IFN-γ-treated EAE mice had significantly less infiltration of inflammatory cells and fewer demyelinated areas. Furthermore, dampening of neuroinflammation by IFN-γ was associated with decreased frequency of CNS infiltrating CD11b^+^ cells and activated MC/MG and increased frequency of resting MG. Decrease of absolute cell number of CD11b^+^ cells was associated with a selective decrease in the absolute cell number of activated CD45^high^ cells without changes in the absolute cell number of resting CD45^low^ cells. This effect could reflect a decreased infiltration of peripheral MC, a deactivation of CD45^high^ activated MC/MG or both processes induced by treatment with IFN-γ. We have found no significant difference in the total numbers of cells in lymph nodes and spleen from IFN-γ- and PBS-treated EAE mice. However, splenic CD11b^+^ cells and neutrophils were significantly reduced in EAE mice treated with IFN-γ; whilst macrophages were not significantly altered. Further studies will be necessary to determine if other peripheral subsets of CD11b^+^ cells are influenced by IFN-γ. In addition, we have found a similar number of cells and frequencies of CD4^+^ T cells, Th1, and Th17 cells in the periphery and the CNS of IFN-γ- and PBS-treated EAE mice (unpublished data). Although IFN-γ did not affect macrophages, these results suggest that IFN-γ might also have a protective role in EAE decreasing the abundance of neutrophils and some other peripheral subset of CD11b^+^ cells, which might indirectly contribute to downregulate MG activation. However, induction of tolerogenic and anti-inflammatory activity in primary MC/MG cultures from the spinal cord of IFN-γ-treated EAE mice by *ex vivo* re-stimulation with low doses of IFN-γ and MOG_35-55_ argues for a direct regulatory role of IFN-γ on these cells. Supporting this view, several studies have demonstrated a direct protective role of IFN-γ in the CNS as well as on MG. Intracerebroventricular (i.c.v.) administration of IFN-γ or intrathecal delivery of an IFN-γ expression system in EAE mice resulted in suppression of clinical symptoms (33, 34). *In vitro* assays have shown that IFN-γ treatment enabled MG to restore homeostasis by promoting neuroprotection, neurogenesis and glutamate clearance (80–82). In turn, i.c.v. injection of IFN-γ-treated MG during the inductive phase of EAE significantly delayed the onset of the disease compared to control mice (83). Remarkably, silencing IFN-γ signaling in MG significantly enhanced EAE severity accompanied by a significant increase in the total number mononuclear cells and in the absolute number of CD11b^+^, CD11b^+^CD45^lo^, CD11b^+^CD45^high^, CD11c^+^, Gr-1^+^, CD4^+^, Th1, and Th17 cells in both spinal cords and brainstems (84). These findings are consistent with our results showing that IFN-γ treatment significantly reduces the total number of mononuclear cells and CD11b^+^ cells in spinal cord of EAE mice and demonstrate that IFN-γ signaling in MG plays an important role in controlling CNS cell infiltration and MG activation. On the other hand, a previous report using bone marrow chimeras showed that the protective effect of IFN-γ in EAE is mediated by an increased production of nitric oxide (NO) at the level of both the periphery and the CNS. Peripheral mononuclear cells were identified as cells producing NO in the periphery whereas the authors suggested that microglia and astrocytes could be involved in the IFN-γ-driven NO production in the CNS (85). Taken together, the evidence suggests that IFN-γ can exert protective effects both in the periphery as in the CNS.

Importantly, immunohistochemistry analysis confirmed a significant increase in the percentage of resting microglia, characterized by a ramified morphology and reduced density and cell size, in response to IFN-γ. Furthermore, high expression of TMEM119, a homeostatic marker of MG, and of MBP1 was observed in the cerebellum from IFN-γ-treated EAE mice, supporting the notion that IFN-γ induces a shift of activated MG to a homeostatic profile. These results are in line with previous observations showing that IFN-γ plays an important role in EAE; regulating inflammation of specific regions of the CNS (55–57, 86).

*Ex vivo* re-stimulation with low doses of IFN-γ (1 ng/ml) and MOG_35-55_ of primary MC/MG cultures obtained from IFN-γ-treated EAE mice resulted in conversion of CD4^+^ T cells into Treg cells associated with higher secretion of TGF-β. Consistently, we have found a significant increase of Treg cells in spinal cord from IFN-γ-treated EAE mice compared to that of the PBS-treated EAE mice (unpublished data). In line with our results, low concentrations of IFN-γ induce a tolerogenic phenotype in MG from neonatal mice, characterized by the expression of intermediate levels of MHC-II and increased secretion of IL-10, capable of inducing Treg cells (59). Supporting our results, a recent study showed that microglia require IFN-γ-signaling to shape the Treg cell compartment in relapsing-remitting EAE and that the absence of microglial IFN-γ-receptor results in worse disease (87). Another study showed that the administration of the microparticle MIS416 in EAE mice induced an IFN-γ-dependent expansion and suppressive function of Treg cells (88). Additionally, a direct role of IFN-γ on the conversion of CD4^+^CD25^-^ T cells to CD4^+^ Treg cells has been reported in EAE (89). Taken together, these results suggest that IFN-γ exerts a tolerogenic role in EAE acting on Treg cells either directly or indirectly through MC/MG.

The contribution of Treg cells to the mechanisms that actively regulate the neuroinflammatory process in EAE has been unequivocally demonstrated (90, 91). Different treatments such as glatiramer acetate, indoleamine 2,3-dioxygenase (IDO), and IL-10 administration, suppress EAE progression promoting an increase in Treg cells (92, 93). In contrast, other treatments such as atorvastatin and trichostatin A suppress EAE progression in a Treg cell-independent manner, suggesting that Treg cells may not always be necessary for the protective effects of some treatments for EAE (94–96). In addition, Korn et al. reported that Treg cells expand in the periphery and accumulate in the CNS but are unable to suppress the proliferation of MOG_35-55_-specific T effector cells from the CNS. Intrinsic resistance of CNS-derived T effector cells to suppression was associated with high production of IL-6 and TNF (97, 98). However, a subsequent report combining targeted depletion of Treg cells with intravital two-photon microscopy concluded that Treg cells mediate recovery from EAE by controlling cytokine production, proliferation, and motility of effector T cells in the CNS (99). Therefore, the activity of Treg cells can be understood as a dynamic process that would depend on the balance between Treg cells and effector T cells as well as the local inflammatory cytokine milieu (90). Additionally, crosstalk between Treg cells and local APC might be critical in modulating effector T cell pathogenicity (100). Our results showing a higher production of TGF-β and conversion of Treg cells in IFN-γ-treated primary MC/MG cultures obtained from IFN-γ-treated EAE mice are consistent with that model of regulation.

We found that MC/MG from EAE mice treated with IFN-γ had a higher expression of CX3CR1, in support of a recent study showing high expression of CX3CR1 in MG at the peak of EAE (10). CX3CR1 is highly expressed in MG (101, 102) as an alert receptor “sensing” the ligand CX3CL1 released by dying neurons. Importantly, lack of this receptor exacerbates inflammation and increases the expression of MHC class II molecules in microglial cells (12–14). In addition, we found that IFN-γ induced a higher frequency of MG with high expression of CX3CR1 and low expression of PD-L1, compared to MG obtained from PBS-treated EAE mice. Although PD-L1 is involved in maintaining immune tolerance and homeostasis through the regulation of T cell activation and differentiation in MS and EAE (103), low expression or absence of PD-L1 has also been related to a tolerogenic effect of APC. Consistent with our findings, low doses of IFN-γ were required to obtain optimal activation of type II macrophages, a subset of macrophages that have been shown to induce a Th2-type anti-inflammatory response after initial activation in an inflammatory environment. Interestingly, IFN-γ-primed type II-macrophages are characterized by an enhanced production of IL-10, reduced expression of IL-12, and low expression of PD-L1, CD40, and CD80. Furthermore, mice receiving IFN-γ-primed type II-activated macrophages were protected from EAE whereas those receiving classically activated macrophages developed EAE (104). In another study, TNF-treated semi-mature DC deficient in PD-L1 showed a stronger tolerogenic capacity in EAE protection compared to wild-type DC. PD-L1^-^/^–^DC-treated EAE mice presented lower numbers of MOG-specific IFN-γ and IL-17 producing cells in the CNS whereas an increased production of the protective cytokines IL-10, IL-13, and IL-4, and reduced levels of IFN-γ and IL-17 were detected in the periphery. Therefore, absence of PD-L1 expression on semi-mature DC enhanced their tolerogenic activity in EAE mice (105).

Bulk RNAseq analysis and recent single-cell RNAseq studies of MG have revealed that unique MG subpopulations, characterized by a distinct signature, emerge during development and homeostasis in the healthy brain as well as during demyelination and remyelination in models of demyelinating and neurodegenerative diseases, including EAE and MS (106–108). These results confirm the ability of MG to shift into different functional states in response to a variety of environmental challenges. Accordingly, our results uncover a new mechanism whereby IFN-γ enables a subset of MG to adapt the transcriptional program into a tolerogenic and anti-inflammatory profile at the peak of EAE. Transcriptional profile analysis of CX3CR1^high^PD-L1^low^ MG isolated from IFN-γ- and PBS-treated EAE mice revealed that genes with a pro-inflammatory role in MC, MG and EAE such as *Cd38* (68–71, 109)*, Cfb* (72, 108)*, Saa3* (75, 76)*, Inhba*, *Anpep* (110, 111), and *Apoc2* (112) were down-regulated by IFN-γ. From these genes, *Cd38, Cfb, Saa3, and Inhba* are considered canonical M1 pro-inflammatory genes in macrophages (73, 74). Transcriptional analysis of MG have showed that genes encoding for apoliprotein C1 and C2 (*Apoc1* and *Apoc2*) were up-regulated during the process of demyelination in the mouse cuprizone model and brain samples from MS patients (107, 112). Interestingly, IFN-γ treatment induced a down-regulation of *Apoc2* in CX3CR1^high^PD-L1^low^ MG. Taken together, these results highlight lipid and lipoprotein metabolism as a key mechanism in the modulation of microglial inflammatory status and as a modifiable target for the treatment of MS (113). A significantly lower frequency and absolute number of activated CX3CR1^high^PD-L1^low^ MG expressing MHC-II molecules was found in IFN-γ-treated EAE mice but not in MG from PBS-treated EAE mice. In addition, a decreased expression of MHC class II antigen presentation gene set was observed in CX3CR1^high^PD-L1^low^ MG from IFN-γ-treated EAE mice compared with CX3CR1^high^PD-L1^low^ MG from control mice; although this difference did not reach statistical significance. Also, *Cfb*, a component of the alternative pathway of complement activation, was downregulated in CX3CR1^high^PD-L1^low^ MG from IFN-γ-treated EAE mice. Consistent with our results, single-cell RNAseq analysis revealed decreased gene expression of the MHC-II antigen presentation pathway both in peripheral APC (dendritic cells, macrophages and B cells) as in microglia in EAE mice treated at the peak of disease with an antigen-specific dual microparticle system (Ag-dMP). In addition, a set of complement genes were downregulated in the microglia from Ag-dMP-treated EAE mice (114). Consequently, EAE mice treated with a monoclonal antibody directed against Cfb significantly attenuated the chronic phase of disease, resulting in reduced cellular infiltration, inflammation and demyelination (72). In line with these results, upregulation in the expression of complement components, including Cfb and MHC-II pathway was determined by single-cell RNAseq analysis in MG isolated during the later stages of neurodegeneration in an Alzheimer’s disease-like animal model (108). Arginase 1 (Arg-1) is an enzyme dominantly expressed in M2 macrophages that hydrolyzes arginine to ornithine and urea, limiting bioavailability of intracellular arginine to be metabolized to NO by the enzyme nitric oxide synthase (NOS), resulting in dampening of inflammation (115, 116). Surprisingly, we found that *Arg1*, encoding arginase 1, was down-regulated in CX3CR1^high^PD-L1^low^ MG by IFN-γ treatment. However, *Nos2*, encoding inducible NOS, was slightly decreased. In addition, *Slc7a2*, which encodes inducible cationic amino acid transporter, and is involved in the uptake of arginine (78), was significantly down-regulated. Consequently, decreased availability of arginine could be expected in response to IFN-γ treatment. Therefore, although *Arg1* is down-regulated in CX3CR1^high^PD-L1^low^ MG, the net result would be an anti-inflammatory effect due to a decrease in the uptake of arginine and decreased *Nos2* expression. This hypothesis is supported by the decreased secretion of nitrites induced by LPS in primary MC/MG cell cultures obtained from IFN-γ-treated EAE mice (**Figure 5D**). On the other hand, *Atp8a2* gene, a selective microglial marker (117), and other genes related to tolerogenic and anti-inflammatory processes in MG and EAE such as DVL-1 (64, 65) and Dusp5 (61, 62) were up-regulated by IFN-γ in CX3CR1^high^PD-L1^low^ MG. Importantly, raising the Log2 Fold change to 1.5, a set of key genes (*Saa3, Inhba*, *Apoc2*, *Atp8a2, and* DVL-1) maintains differential expression in response to IFN-γ treatment.

Our results show that IFN-γ is unable to promote amelioration of EAE symptoms and induction of CX3CR1^high^PD-L1^low^ MG in the absence of STAT-1, indicating that STAT-1 is critical in the protective effects mediated by IFN-γ in EAE. In line with these results, we found that from all up- and down-regulated genes in CX3CR1^high^PD-L1^low^ MG by IFN-γ, 8 of them (*Cfb, Dusp5, Anxa4, Neurl1b, C3, Naca, Anxa11, Slc15a2*) are also regulated by STAT-1, suggesting that the IFN-γ/STAT-1 signaling axis would be involved in suppressing neuroinflammation in EAE regulating the expression of a set of genes involved in microglial activation. Supporting this potential mechanism, previous studies have reported that the IFN-γ/STAT-1 axis regulates the expression of indoleamine 2,3-dioxygenase (IDO), a tryptophan catabolizing enzyme involved in immune tolerance and suppression of EAE (118, 119), in microglia (120). Similarly, the IFN-γ/STAT-1 axis also regulates the tolerogenic activity of IDO in dendritic cells in a mouse model of prediabetes (121).

To obtain more detailed insights into the molecular interactions between the differentially expressed genes in MG in response to IFN-γ, we created a molecular network of protein-protein interactions based on the known interactions between the products of the genes targeted by IFN-γ. In this analysis, the number of interacting genes depended on the existing knowledge available from the STRING database and does not necessarily reflect the actual number of biological interactions. Therefore, this model is biased as some genes have been widely studied by many investigators, others less so. Despite this, the model reveals molecules physiologically interacting with those identified by expression profiling and delivers a more complete understanding of their connectedness. These analyses highlight the master role that IFN-γ plays in regulating microglial activity and provide new insights into the cellular and molecular mechanisms involved in the therapeutic activity of IFN-γ in EAE.

## 5 Conclusions

Our findings show that IFN-γ exerts therapeutic activity in EAE by regulating myeloid cell infiltration and inducing attenuation of neuroinflammation and a shift from activated MG to resting MG. In addition, IFN-γ promotes the induction of homeostatic CX3CR1^high^PD-L1^low^ MG, characterized by a homeostatic and anti-inflammatory transcriptional signature. The amelioration of clinical symptoms and the induction of CX3CR1^high^PD-L1^low^ MG were dependent on STAT-1. Also, our analyses reveal that IFN-γ plays a master role in regulating a network of genes involved in microglial activation. Taken together, our findings uncover a novel cellular and molecular mechanism whereby IFN-γ exerts therapeutic activity in EAE and contribute to clarify the complex role that IFN-γ plays in EAE and MS.

## Data availability statement

The RNA sequencing datasets generated for this study have been deposited in the NCBI GEO database under accession number GSE231833.

## Ethics statement

The animal study was reviewed and approved by the Institutional Animal Care and Use Committee (IACUC) of the University of Chile and Northwestern University.

## Author contributions

JT and RN conceived the project. JT, EA, GA, LG, PM, CP, JI-V, and EL performed the experiments and analysis of the data. MR and NK performed RNA-seq analysis. PG-H performed molecular network analysis and its interpretation. RN, JT, PG-H, PIB, SM discussed the results and wrote the manuscript. All authors contributed to the article and approved the submitted version.
