# Supplementary material for: Interferon-gamma ameliorates experimental autoimmune encephalomyelitis by inducing homeostatic adaptation of microglia
Source: Front Immunol. 2023 Jun 2;14:1191838. doi: 10.3389/fimmu.2023.1191838 (PMC10272814; doi:10.3389/fimmu.2023.1191838)
Supplement: Supplementary file 1 [file DataSheet_1.pdf]

Supplementary Material

Interferon-gamma ameliorates experimental autoimmune encephalomyelitis by inducing homeostatic adaptation of microglia.

Juan E. Tichauer[1]†, Gabriel Arellano[1,5]†, Eric Acuña[1], Luis F. González[1], Nirmal R. Kannaiyan[2], Paola Murgas[3], Concepción Panadero-Medianero[3], Jorge Ibañez-Vega[1], Paula I. Burgos[4], Eileah Loda[5], Stephen D. Miller[5], Moritz J. Rossner[2], Peter J. Gebicke-Haerter[1,6], Rodrigo Naves[1*].

*Correspondence: rodrigonaves@uchile.cl

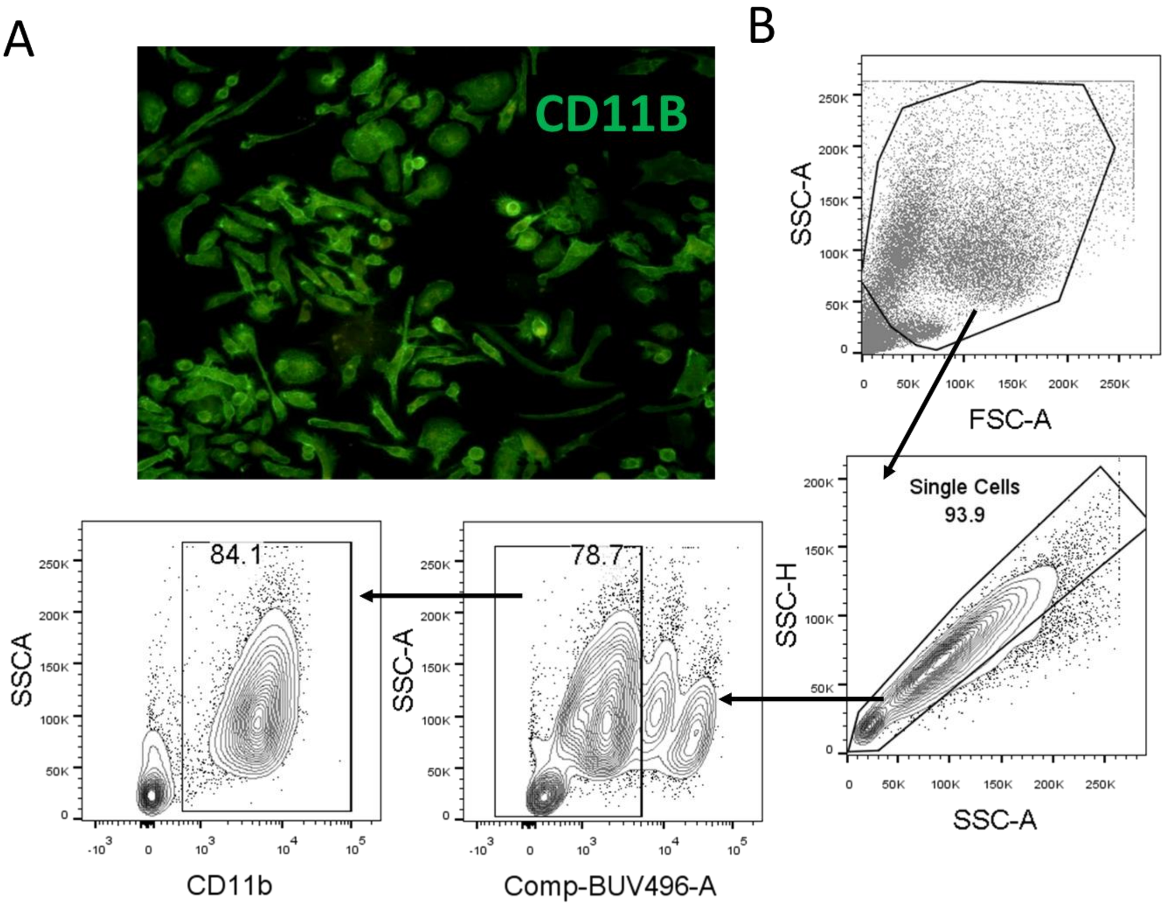

Figure S1. **Primary adherent mononuclear cell cultures established from spinal cords of EAE mice were mainly composed of resting CD11b$^+$ cells (MC/MG). (A)** Primary adherent mononuclear cell cultures obtained from spinal cords of EAE mice were immunostained for CD11b (green) and analyzed by immunofluorescence microscopy. Cells were mainly composed of CD11b$^+$ cells (MC/MG), and exhibited a resting phenotype characterized by an elongated shape in contrast to the "fried egg" shape morphology of activated cells. **(B)** Flow cytometry analysis confirmed that most of the primary adherent mononuclear cell cultures were CD11b$^+$ (80-90%). Cell viability was assessed using the Zombie UV fluorescent dye (Comp-BUV496).

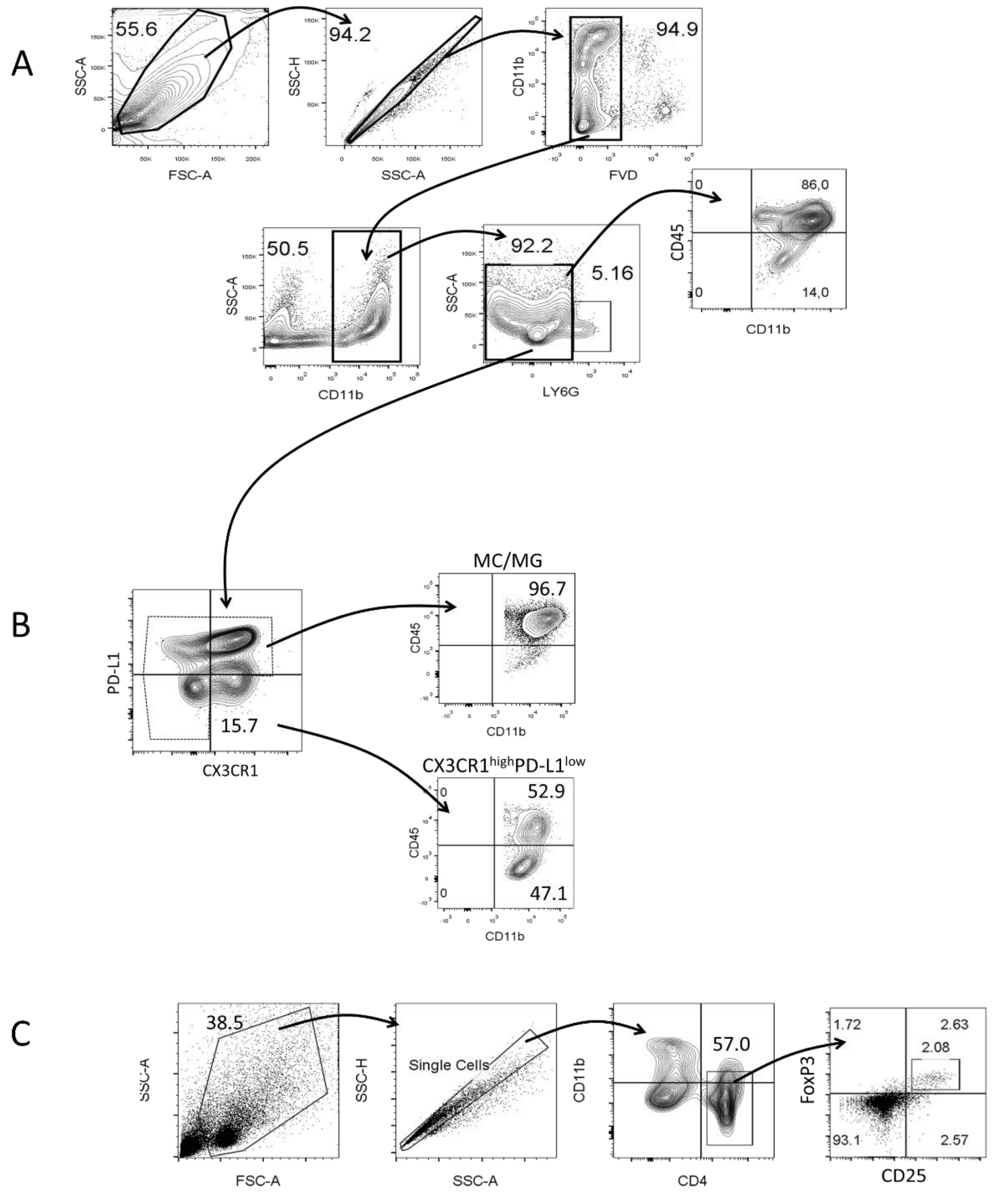

Figure S2. **Flow cytometry gating strategy.** For data analysis the following gating strategies were followed in sequence. For all analysis, immune cell populations were first gated based on their scatter properties (FSC-A and SSC-A), and doublets were excluded by gating on side scatter height (SSC-H) vs side scatter area (SSC-A). Dead cells were excluded by analysis of fixable viability dye (FVD) staining. Subsequent phenotypic analyses were performed to identify a population of myeloid cells/microglia (MC/MG) ($CD11b^{+}Ly6G^{-}$) and neutrophils ($CD11b^{+}Ly6G^{+}$) **(A)**. In other assays, a subset of microglia (MG) was identified as $CX3CR1^{high}PD\text{-}L1^{low}$ gated on $CD11b^{+}Ly6G^{-}$ **(B)**. In the population MC/MG ($CD11b^{+}Ly6G^{-}$) and $CD11b^{+}Ly6G^{-}CX3CR1^{high}PD\text{-}L1^{low}$ MG, activated and resting cells were identified as $CD45^{high}$ and $CD45^{low}$, respectively. Gating strategy for analysis of Treg cells in the co-culture of MC/MG ($CD11b^{+}Ly6G^{-}$) and purified $CD4^{+}$ T cells consisted of selection of $CD4^{+}$ T cells followed by a gate on $CD25^{high}$ and $FoxP3^{+}$ **(C)**. The numbers in the plots indicate the percent of positive cells in each gate or quadrant.

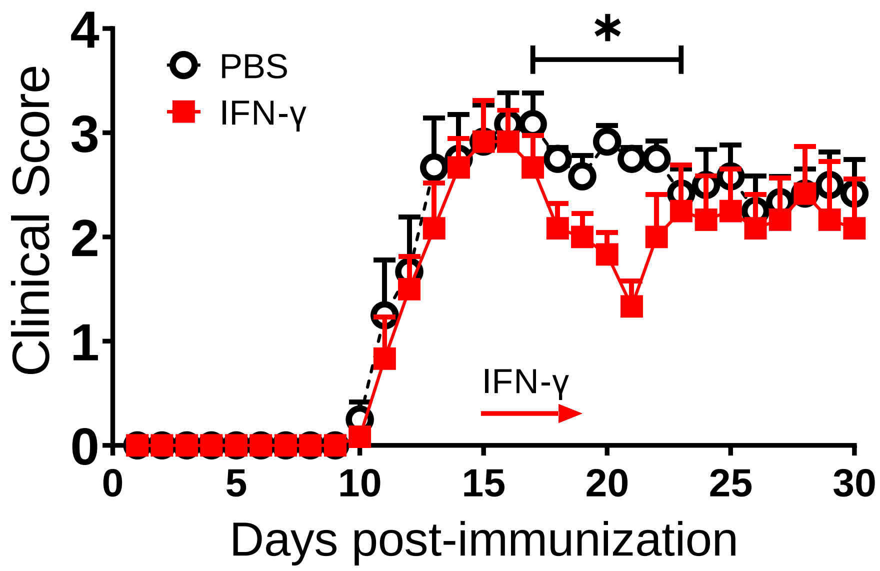

Figure S3**. IFN-γ treatment starting at the peak of EAE ameliorates severity of clinical symptoms of EAE.** EAE mice were treated at the peak of disease with 1 μg IFN-γ (red squares) or PBS (black circles) for 5 days, and monitored daily until day 30 post-immunization. Error bars represent mean ± SEM. n= 6 mice per group. **P*<0.05.

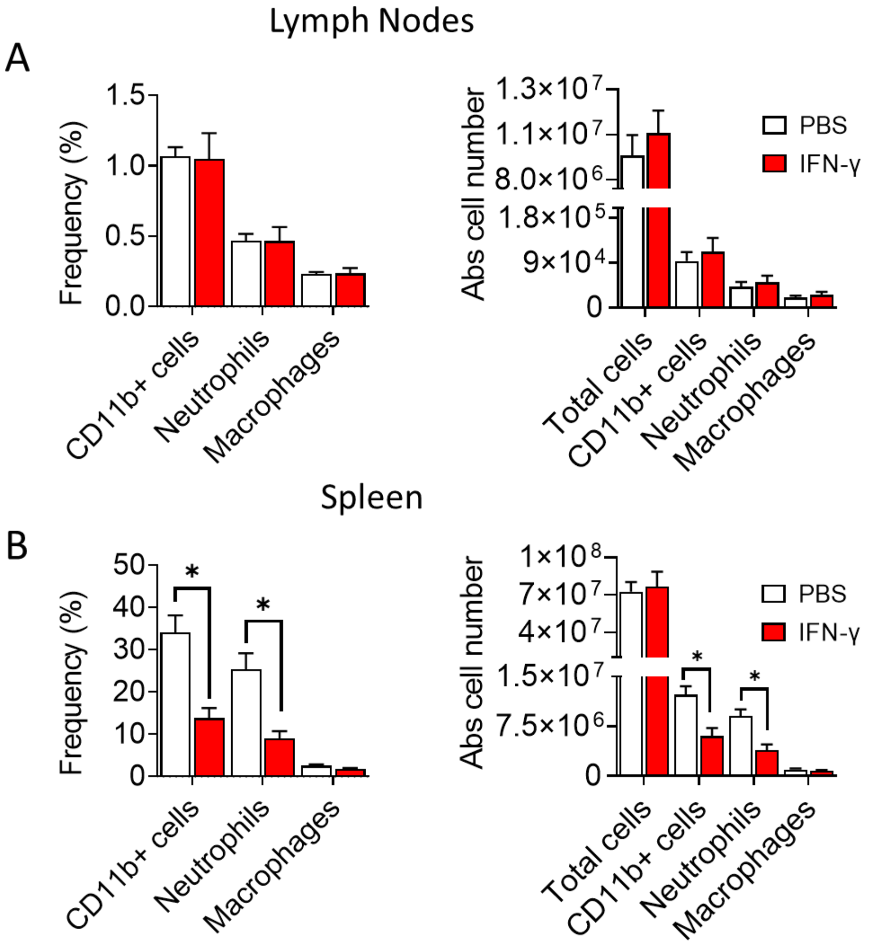

Figure S4. **Effect of IFN-γ treatment on myeloid cells in secondary lymphoid organs.** A suspension of cells isolated from **(A)** draining lymph nodes and **(B)** spleen of EAE mice treated with either PBS (white bar) or 1 µg IFN-γ (red bar) for 5 days at the peak of EAE were used to determine the absolute cell number and the frequency of $CD45^+CD11b^+$ cells, neutrophils ($CD45^+CD11b^+Ly6G^+$), and macrophages ($CD45^+CD11b^+F4/80^+$) by flow cytometry. n= 4-5 mice per group. Results are shown as the mean ± SEM. * $P < 0.05$.

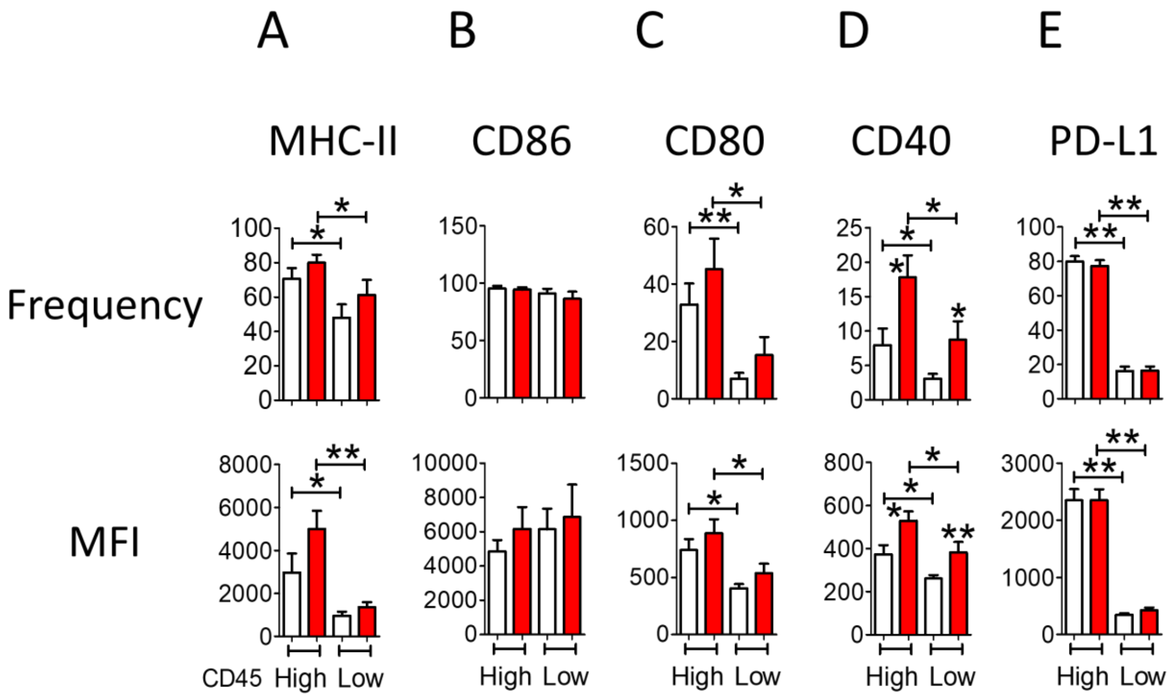

Figure S5. **Expression of MHC-II, co-stimulatory, and co-inhibitory molecules in activated MC/MG and resting MG cells**. Fresh mononuclear cells obtained from the spinal cords of EAE mice treated with PBS (white bars) or IFN-γ (red bars) were analyzed for the expression of MHC-II, co-stimulatory, and co-inhibitory molecules by flow cytometry. **(A-E)** Frequency and mean of fluorescence intensity (MFI) of activated MC/MG ($CD11b^{+}Ly6G^{-}CD45^{high}$) and resting MG ($CD11b^{+}Ly6G^{-}CD45^{low}$) cells expressing **(A)** MHC-II molecules, **(B)** CD86, **(C)** CD80, **(D)** CD40 and **(E)** PD-L1. n= 5 mice per group; 5 independent experiments. Results are shown as the mean ± SEM. * $P < 0.05$; ** $P < 0.01$.

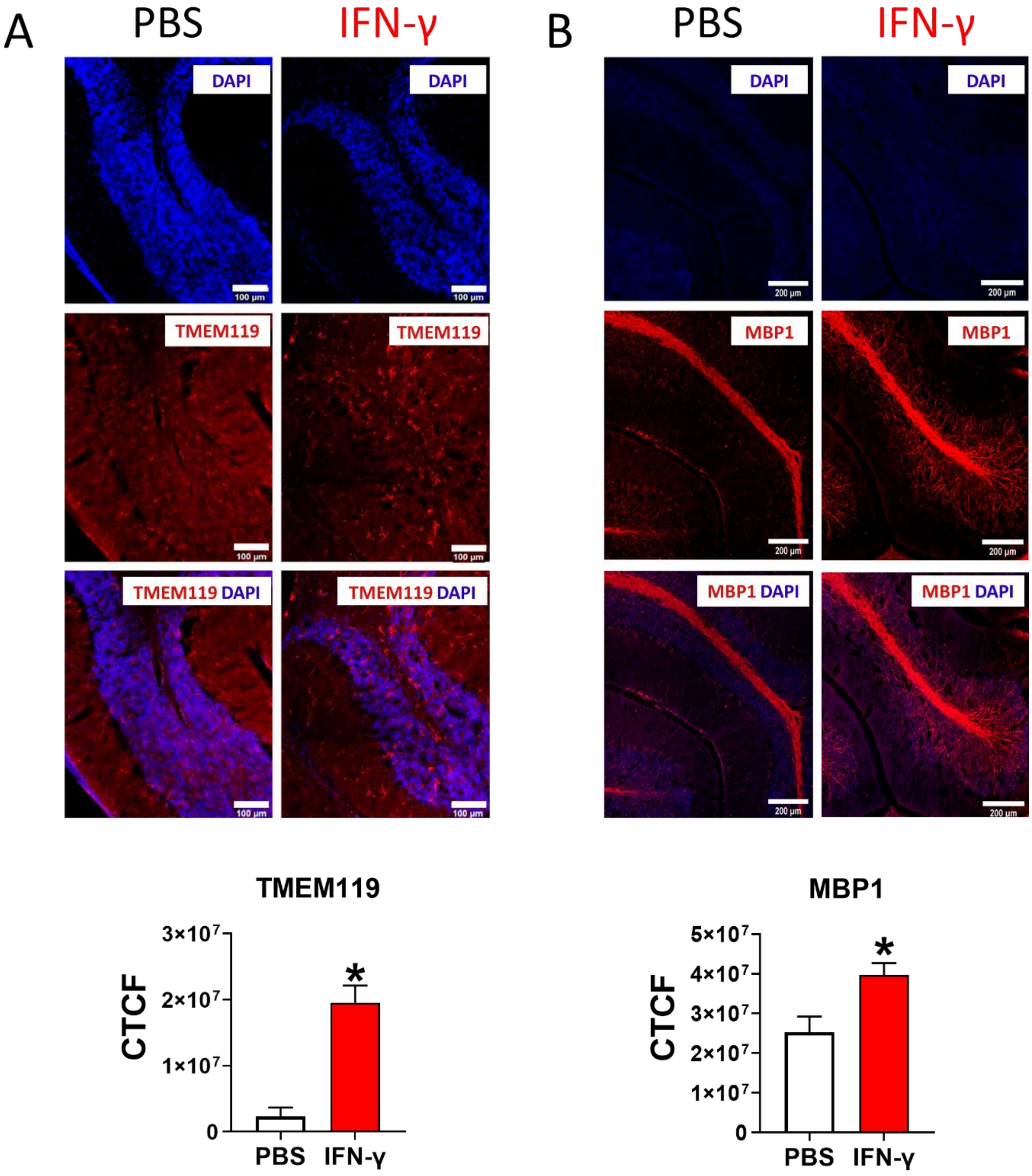

Figure S6. **Expression of TMEM119 and MBP1 is increased in the cerebellum from IFN-γ-treated EAE mice.** Cerebellums obtained from EAE mice treated with IFN-γ or PBS were subjected to immunostaining against **(A)** TMEM119 (red) and **(B)** MBP1 (red). Cell nuclei were stained using Dapi (blue). The magnification bar represents 100 µm in the left panels and 200 µm in the right panels. Quantification of fluorescence intensity for TMEM119 and MBP1 are shown as the mean ± SEM. CTCF= Corrected total cell fluorescence. n= 6 mice.

Method S1: Fixation and tissue processing for TMEM and MBP1 immunostaining. Cerebellum obtained from IFN-γ and PBS treated mice were fixed in 4 % paraformaldehyde in PBS pH=7.4 at 4°C for 3 h. Then, tissues were immersed in a sterile sucrose gradient (10-30%) in PBS and stored at 4°C until further use. Coronal sections of tissues were cut to a thickness of 12 µm. Glass-mounted tissues were immunostained. Briefly, tissue slides were permeabilized with TBS 0.3% triton (Perm solution) for 30 min and blocked with 5 % BSA in perm solution for 1 h. Primary antibody was applied diluted in TBS 0.3% triton 5% BSA (1:100 rabbit TMEM119, Abcam, Cambridge, MA, US) or TBS 0.3% triton 5% BSA 1:70 (mouse MBP1, Biolegend, San Diego, CA, US) at 4°C, overnight. The next day, slides were washed 3 times for ten minutes each in TBS buffer, and secondary antibody was applied (1:1000 anti-rabbit Alexa Fluor555, Invitrogen, Waltham, MA, US or 1:500 or anti-mouse Alexa Fluor555, Invitrogen, Waltham, MA, US). Slides were washed 3 times for ten minutes each in TBS buffer, and cell nuclei were stained with DAPI (blue). Finally, slides were rinsed 1 time with TBS, mounted with an anti-fade mounting media and visualized in a Leica DMI8 inverted fluorescence microscope. Image fluorescence was analyzed using Image J software (NIH, USA). The fluorescence was expressed as corrected total cell fluorescence (CTCF), which is the integrated density minus the area of selected cell x mean fluorescence of background readings.

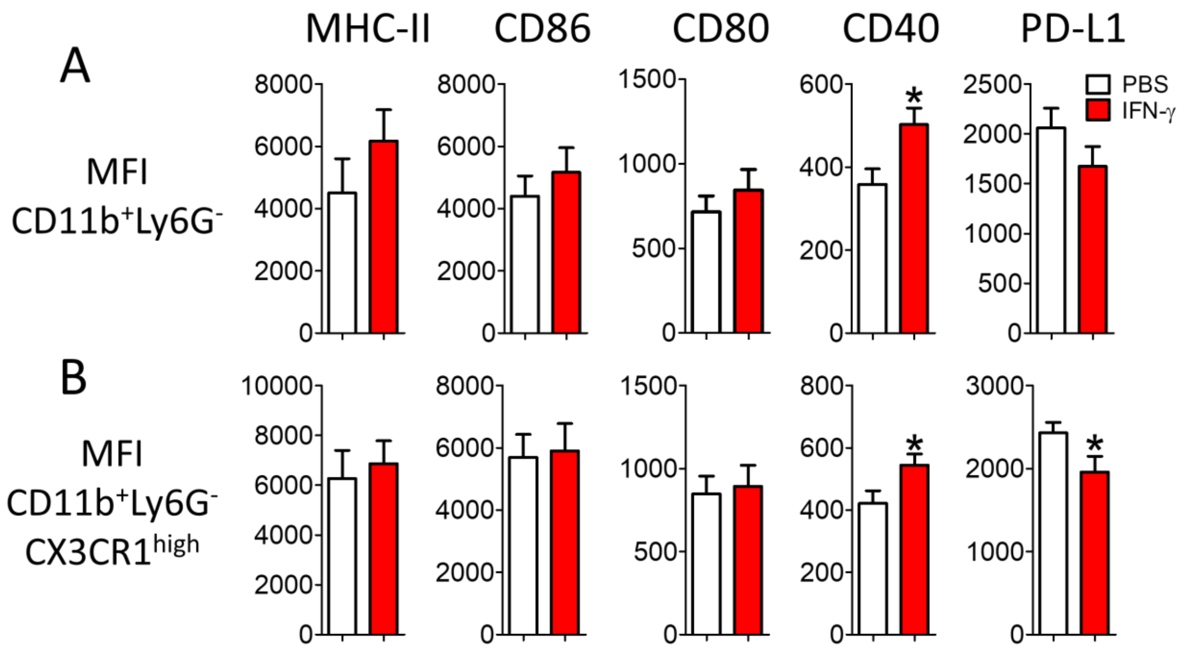

Figure S7. **Expression of MHC-II, co-stimulatory, and co-inhibitory molecules in $CD11b^{+}Ly6G^{-}$ cells (MC/MG) and $CX3CR1^{high}CD11b^{+}Ly6G^{-}$ cells.** Fresh mononuclear cells from the spinal cords of EAE mice treated with PBS (white bars) or IFN-γ (red bars) were analyzed for MHC-II, CD86, CD80, CD40, and PD-L1 by flow cytometry in **(A)** $CD11b^{+}Ly6G^{-}$ and **(B)** $CD11b^{+}Ly6G^{-}CX3CR1^{high}$ subpopulations. n= 5 mice per group; 5 independent experiments. Results are shown as the mean ± SEM. * $P < 0.05$. MFI: Mean of fluorescence intensity.

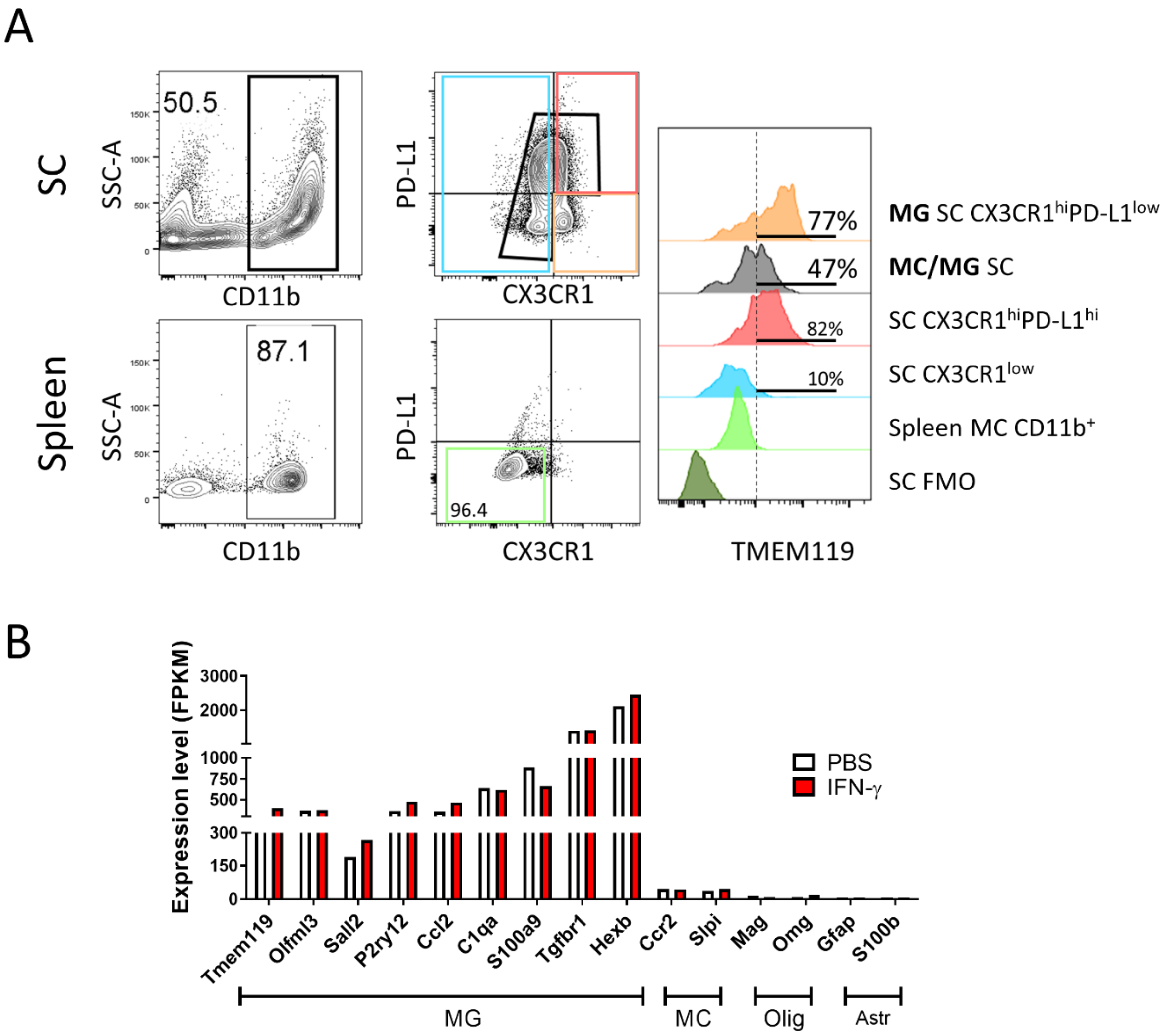

Figure S8. **Characterization of CX3CR1^{high} PD-L1^{low} MG.** Mononuclear cells obtained from SC of EAE mice treated with PBS for 5 days during the late effector phase were analyzed by flow cytometry. **(A)** CX3CR1^{high} PD-L1^{low} MG gated in CD11b^{+}Ly6G^{-} were analyzed for the expression of TMEM119. CD11b^{+} cells isolated from the spleen were used as a negative control. FMO: Fluorescence minus one. **(B)** CX3CR1^{high} PD-L1^{low} MG subset was purified from SC of EAE mice treated with PBS (white bars) or IFN-γ (red bars) by cell sorting and gene expression profile analyzed by RNA-seq. The expression of gene markers for microglia (MG), myeloid cells (MC), oligodendrocytes (Olig) and astrocytes (Astr) is shown.

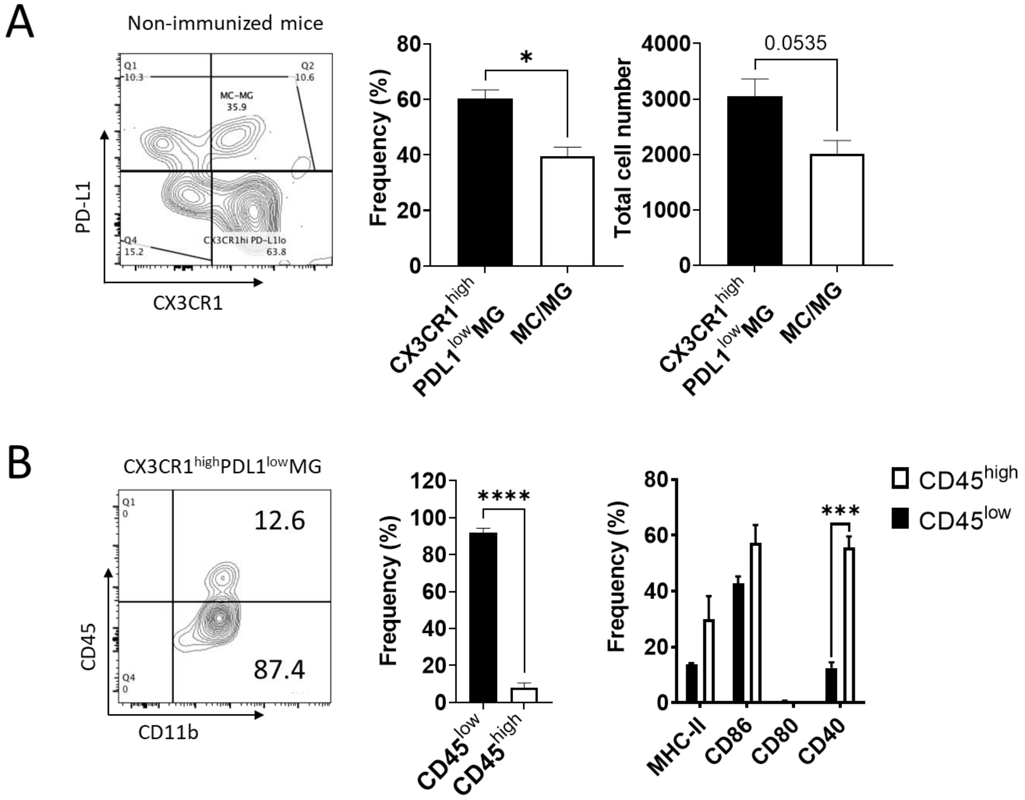

Figure S9. **The frequency of CD11b$^+$Ly6G$^-$CX3CR1^{high}PDL1^{low} MG is higher than the frequency of MC/MG cells in the spinal cords of non-immunized mice**. Mononuclear cells from SC of non-immunized WT mice were used to determine **(A)** the frequency and absolute cell number of CD11b$^+$Ly6G$^-$CX3CR1^{high}PDL1^{low}MG and MC/MG. **(B)** Frequencies of activated (CD11b$^+$CD45^{high}) and resting (CD11b$^+$CD45^{low}) CX3CR1^{high}PD-L1^{low} MG expressing MHC-II molecules and co-stimulatory molecules (CD80, CD86, and CD40).

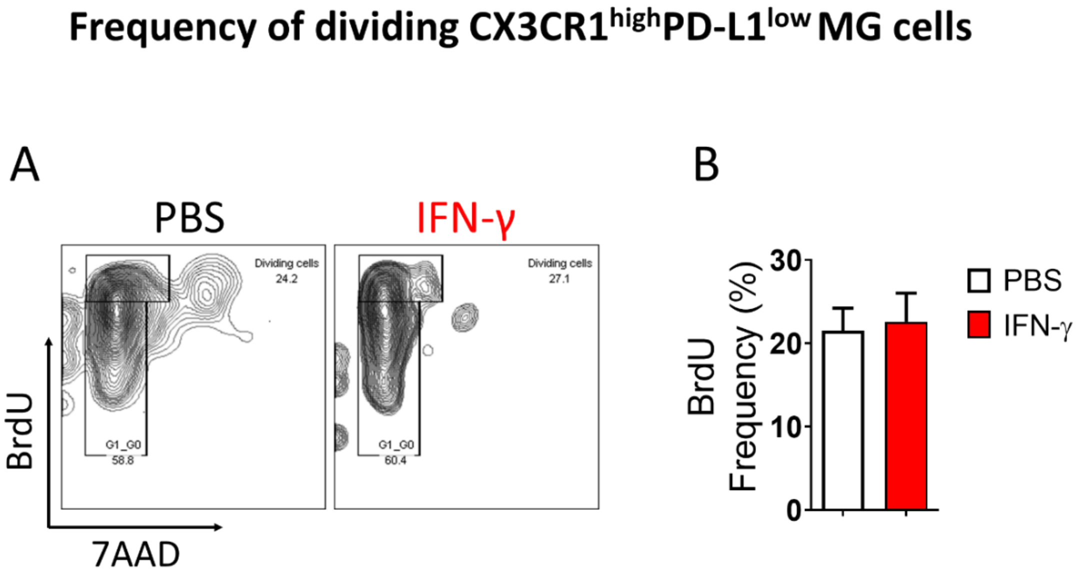

Figure S10. **Spinal cord CX3CR1^{high}PD-L1^{low} MG cell proliferation is not increased by IFN-γ treatment.** EAE mice treated with IFN-γ or PBS received a simultaneous i.p. injection of 100 µl 5-bromo-2´-deoxyuridine (BrdU) (1 mg/ml) for 5 days during the late effector phase. Mononuclear cells were isolated from SC of EAE mice and BrdU and 7AAD incorporation was analyzed by flow cytometry. **(A)** Representative flow cytometry analysis of gated CX3CR1^{high}PDL1^{low} cells to determine the frequency of BrdU^{+} cells. **(B)** Frequency of CX3CR1^{high}PDL1^{low} cells BrdU^{+} from EAE mice treated with PBS (white bars) or IFN-γ (red bars). n= 6 mice. Results are shown as the mean ± SEM.

Method S2. *In vivo* cell proliferation. EAE mice treated with IFN-γ or PBS received a simultaneous i.p. injection of 100 µl of 5-bromo-2´-deoxyuridine (BrdU) (1 mg/ml) (Sigma, Saint Louis, Missouri, US) in PBS daily for 5 days during the late effector phase. Mononuclear cells were isolated from SC using 40%/70% discontinuous Percoll gradients (Amersham, Piscataway, New Jersey, US) as described in methods, section 2.4. Live cells were labeled by Zombie-UV (Biolegend, San Diego, CA, US), following manufacturer instructions. Then, cells were extracellularly labeled for Ly6G-BV605 (Biolegend, San Diego, CA, US), CD11b-BUV395 (BD Biosciences, San Jose, CA, US), CX3CR1-PE/Cy7 (Biolegend, San Diego, CA, US), PD-L1-BV711 (Biolegend, San Diego, CA, US) in 100 µL of PBS 2%FCS (FACS buffer) for 1 hour at 4°C. Cells were washed with FACS buffer and centrifuged at 400 g x 5 min. The cell pellet was resuspended in 100 µL of Foxp3/transcription factor fixation buffer (Invitrogen, Waltham, MA, US) and incubated at 4°C for 15 min. Next, cells were washed with FACS buffer and resuspended in 100 µL Foxp3/transcription factor permeabilization buffer (Invitrogen, Waltham, MA, US), and incubated at room temperature for 10 min. Next, cells were washed with FACS buffer and resuspended in PBS supplemented with 1mM CaCl2 and 0.5mM MgCl2, adding 5 units of DNAse1 and incubated at 37° for 1 hour. Next, cells were washed with FACS buffer and fixed overnight at 4°C. Then, cells were washed and resuspended in 100 µL of permeabilization buffer and incubated with anti-BrdU (Biolegend, San Diego, CA, US) for 1 hour at 4°C. Cells were washed with permeabilization buffer and resuspended in 100 µL PBS with 7-AAD (Biolegend, San Diego, CA, US). Finally, BrdU and 7AAD incorporation was analyzed using a Fortessa-X20 Flow Cytometry (BD Biosciences, US) and FlowJo software (Tree Star, US).
